# A xenograft and cell line model of SDH-deficient pheochromocytoma derived from Sdhb+/− rats

**DOI:** 10.1530/ERC-19-0474

**Published:** 2020-04-03

**Authors:** James F Powers, Brent Cochran, James D Baleja, Hadley D Sikes, Andrew D Pattison, Xue Zhang, Inna Lomakin, Annette Shepard-Barry, Karel Pacak, Sun Jin Moon, Troy F Langford, Kassi Taylor Stein, Richard W Tothill, Yingbin Ouyang, Arthur S Tischler

**Affiliations:** 1 Department of Pathology and Laboratory Medicine, Tufts Medical Center, Tufts University School of Medicine, Boston, Massachusetts, USA; 2 Department of Developmental, Molecular and Chemical Biology, Tufts University School of Medicine, Boston, Massachusetts, USA; 3 Department of Chemical Engineering, Massachusetts Institute of Technology, Cambridge, Massachusetts, USA; 4 Department of Clinical Pathology, University of Melbourne, Melbourne, Victoria, Australia; 5 Section on Medical Neuroendocrinology, Eunice Kennedy Shriver Division National Institute of Child Health and Human Development, Bethesda, Maryland, USA; 6 Peter MacCallum Cancer Centre, Melbourne, Victoria, Australia; 7 Cyagen US Inc, Santa Clara, California, USA

**Keywords:** succinate dehydrogenase B, paraganglioma, pheochromocytoma, cluster 1, xenograft, cell culture, hypoxia, metabolome, model, rat

## Abstract

Tumors caused by loss-of-function mutations in genes encoding TCA cycle enzymes have been recently discovered and are now of great interest. Mutations in succinate dehydrogenase (SDH) subunits cause pheochromocytoma/paraganglioma (PCPG) and syndromically associated tumors, which differ phenotypically and clinically from more common SDH-intact tumors of the same types. Consequences of SDH deficiency include rewired metabolism, pseudohypoxic signaling and altered redox balance. PCPG with *SDHB* mutations are particularly aggressive, and development of treatments has been hampered by lack of valid experimental models. Attempts to develop mouse models have been unsuccessful. Using a new strategy, we developed a xenograft and cell line model of SDH-deficient pheochromocytoma from rats with a heterozygous germline *Sdhb* mutation. The genome, transcriptome and metabolome of this model, called RS0, closely resemble those of *SDHB*-mutated human PCPGs, making it the most valid model now available. Strategies employed to develop RS0 may be broadly applicable to other SDH-deficient tumors.

## Introduction

Paraganglia are neuroendocrine structures that develop from neural crest progenitors associated with paraxial sympathetic nerves and from parasympathetic nerves in the head and neck. The major sympathetic paraganglion is the adrenal medulla. Tumors called paragangliomas can arise anywhere in the distribution of normal paraganglia. By definition an intra-adrenal paraganglioma (PG) is called a pheochromocytoma (PC) (Lloyd* et al.* 2017).

At least 40% of pheochromocytomas and paragangliomas (PCPGs) are hereditary, and germline mutations of at least 17 functionally diverse genes can lead to their development (Dahia 2017). Mutations of genes encoding subunits of succinate dehydrogenase (SDH) account for the largest number of familial aggregates of these tumors and for more than 20–30% of the tumors that metastasize (Amar* et al.* 2005, 2007, Benn* et al.* 2006, Pasini & Stratakis 2009, Favier* et al.* 2015). Mutations of *SDHB,* which encodes the B subunit, confer the highest risk of metastasis (Crona* et al.* 2019, Hescot* et al.* 2019). Specifically, about 42% of *SDHB* mutation carriers develop pheochromocytomas or paragangliomas by the age of 70 (Jochmanova* et al.* 2017, Rijken* et al.* 2017), and up to almost 60% of the tumors have been reported to metastasize (Jochmanova* et al.* 2017). There is currently no cure after metastases occur. Further, there are few experimental models for pre-clinical testing of new treatments and no models that faithfully reflect the tumor phenotype.

Paragangliomas sort into clusters based on their transcriptome and associated phenotypic characteristics, which include signaling pathways, metabolism and hormonal function (Dahia* et al.* 2005, Richter* et al.* 2014, Castro-Vega* et al.* 2015, Favier* et al.* 2015, Fishbein* et al.* 2017, Fishbein & Wilkerson 2018). Tumors with gene mutations of SDH subunits fall into cluster 1, which is characterized by pseudohypoxic signaling and metabolism (Eisenhofer* et al.* 2017). In contrast, cluster 2, the second major aggregate of tumors, is associated predominantly with mutations of genes including *RET* and *NF1* that affect kinase signaling. It is important for pre-clinical models to represent the appropriate tumor cluster as closely as possible because distinctive cluster-associated signaling and metabolic (Richter* et al.* 2014, Lussey-Lepoutre* et al.* 2015) characteristics can potentially serve as drug targets (Tella* et al.* 2017) or modify drug responses (Muir & Vander Heiden 2018). However, genetically engineered mice with *Sdhb* mutations have thus far failed to develop SDH-deficient PCPGs. Consequently, cluster 2 mouse tumor models are often used as imperfect surrogates (Lussey-Lepoutre* et al.* 2018).

In contrast to mice and other species, rats have an unusual proclivity to develop PCs. The incidence of these tumors increases throughout life and is especially high in males compared to females, with a ratio up to 10:1 in some rat strains (Tischler* et al.* 2014). Abdominal or head and neck PGs are also occasionally reported (van Zwieten* et al.* 1979, Hall* et al.* 1987, Li* et al.* 2013). In WT Sprague–Dawley rats, PCs begin to be detected at age 1–2 years, and the prevalence at 2 years is approximately 10% (Pace* et al.* 2002). The incidence is further increased by a variety of agents including high calorie diet, radiation, drugs and hormones (Tischler* et al.* 2004, 2014). In view of their innate proclivity to develop PCCs, we hypothesized that rats would *a priori* be superior to mice as a potential *Sdhb* tumor model. This communication describes the successful development of a new rat-derived xenograft and cell culture model that closely mirrors the characteristics of *SDHB*-associated human PCPGs.

## Materials and methods

### Origin and validation of the model

Heterozygous *Sdhb*^+/-^ Sprague–Dawley rats were generated by a commercial supplier (Cyagen Biosciences, Santa Clara, CA, USA) using the TALEN (transcription activator-like effector nuclease) technique (Merkert & Martin 2018). Four ‘F0’ founders were generated with various deletions in the rat *Sdhb* gene: a 1-base deletion in exon 1, a 13-base deletion in exon 1, a 7-base deletion in exon 2 and a 8-base deletion in exon 2. All of the mutations except the 1-base deletion successfully became germline on subsequent breeding. For each of the founder mutations, we tested liver tissue of heterozygous offspring for expression of rat Sdhb mRNA using a commercial TaqMan assay kit (Life Technologies). Enzymatic assays of liver tissue were also performed (Abcam Complex ll Enzyme Activity Microplate Assay Kit). Finally, as a bioassay to confirm biological significance of the *Sdhb* deletions, we bred male and female *Sdhb*^+/-^ heterozygotes to each other and examined gravid uteri as a test for homozygous embryonic lethality. Sperm from rats with all three germline deletions is cryopreserved at the Rat Resource and Research Center (RRRC) at the University of Missouri (rrrc@missouri.edu), where it will be publicly available.

### Development and harvesting of tumors

Thirty-five male *Sdhb*^+/-^ rats with a 13-bp deletion in exon 1 (Supplementary Fig. 1, see section on supplementary materials given at the end of this article) formed the core of this study. Twenty-one of the animals were exposed to 5 Gy of gamma irradiation 1 week postnatally. The administration of postnatal irradiation was based on previous findings that irradiation of the parent animals contributed to the development of the rat-derived PC12 (Warren & Chute 1972) and mouse-derived MPC (Powers* et al.* 2000) PC cell lines. All were fed *ad libitum* on a standard rodent diet (Envigo/Harlan, Indianapolis, IN, USA) and were maintained until they were killed because of deteriorating health or were found dead. Necropsies were performed and the adrenal glands, pituitaries and carotid bodies were examined grossly and microscopically. Tissue from five PCs that appeared to be viable was injected subcutaneously into NSG mice as previously described (Powers* et al.* 2017). Xenografts of the PC12 rat pheochromocytoma cell line (passage 40), (Greene & Tischler 1976) were similarly prepared to serve as controls in relevant analyses.

Primary tumors and xenografts in consecutive passages were examined histologically and immunohistochemically as previously reported (Powers* et al.* 2018). Antibodies utilized are shown in Supplementary Table 1. Immunoblots and electron microscopy were also performed as previously described (Powers* et al.* 2018).

### Cell cultures and development of cell lines

To generate primary cell cultures, minced xenograft tissue was dissociated in collagenase followed by trypsin (Powers* et al.* 2000). The dissociated cell suspension was plated in 35 mm culture dishes in a series of preliminary studies to test the effects of varied medium composition and O_2_ concentration in attempting to establish cell lines. The utilization of xenografts to develop cell lines, rather than vice versa, served to expand tumor cell populations because the usually small primary tumors did not provide sufficient numbers of cells for adequate tests of growth conditions. It further provided xenografts comparable to human patient-derived xenograft (PDX) models, which are not derived from cell lines. This approach has been utilized by others for studies of human neuroblastomas (Persson* et al.* 2017).

Routine culture medium consisted of RPMI 1640 with 10% heat inactivated horse serum, 5% fetal bovine serum, glutamine and penicillin/streptomycin. This medium is employed in our laboratory to culture PC12 cells (Greene & Tischler 1976), normal rat chromaffin cells (Tischler* et al.* 1994) and mouse-derived MPC cells (Powers* et al.* 2000). Because SDH deficiency alters cells’ nutritional requirements (Lussey-Lepoutre* et al.* 2015), we compared cell growth and survival in this medium vs an enriched medium supplemented with non-essential amino acids, fatty acids, uridine and pyruvate that we recently employed for primary cultures of a SDH-deficient gastrointestinal stromal tumor (GIST) (Powers* et al.* 2018). These basal and enriched media were compared to serum-free stem cell media consisting either of DMEM/F12 with B27 Supplement plus FGF (20 ng/mL) and EGF (20 ng/mL) or serum-free RPMI 1640 with the same B27 and growth factor supplementation. Also, based on the gastrointestinal stromal tumor study, which showed deleterious effects of O_2_ on cell survival, we tested the effects of maintaining cultures in decreased oxygen concentrations (10%, 5% or 1%) compared to traditional ‘normoxic’ cultures (~20% O_2_, 95% air/5% CO_2_). In order to simultaneously compare multiple O_2_ concentrations, cultures were maintained in Billups-Rothenberg modular incubator chambers with pre-mixed gasses with increased N_2_ to compensate for decreased O_2_. CO_2_ concentration was constant at 5%_._ All cultures were maintained in a water-saturated atmosphere at 37°C. Double immunocytochemical staining for tyrosine hydroxylase (TH) and incorporation of bromodeoxyuridine (BrdU) (Tischler* et al.* 1992) was performed in order to distinguish the neoplastic chromaffin cells from fibroblasts, endothelial cells and other normal cell types. Two cell lines, designated RS0 (for rat *Sdhb* null) and RS1/2 (for *Sdhb* haploinsufficient), originated from cultures of xenografts derived from two different primary tumors and maintained in 5% O_2_.

### Whole genome sequencing

#### DNA extraction

Cell line DNA was extracted using a Qiagen Blood and Cell Culture DNA kit (catalogue number 13362) according to the manufacturer’s instructions. Matched normal DNA was extracted from formalin-fixed, paraffin-embedded sections of kidneys from the individual rats in which the primary RS0 and RS1/2 tumors originated, using the Qiagen DNeasy Blood and Tissue kit (catalogue number: 69504) with a slightly modified protocol. Samples were deparaffinized in xylene and subjected to an additional ethanol wash to remove residual xylene. Proteinase K digestion was carried out at 56°C over 3 days instead of overnight, with 12 µL proteinase K added every 24 h. The optional RNase A treatment was also carried out using 4 µL of RNase (100 mg/mL) at the end of the 3-day incubation.

#### WGS library preparation and sequencing

Libraries were prepared at The University of Melbourne Centre for Cancer Research (UMCCR) using the Illumina® TruSeq™ DNA Nano library preparation method according to the manufacturer’s instructions. Two hundred nanograms of DNA was used as input and a 550-bp insert size was targeted. Samples were sequenced in separate batches on the Illumina® Nova-Seq 6000. QC stats including the mean read count and insert size from each sample can be seen in Supplementary Table 2.

#### WGS alignment and quality control

BCL files were demultiplexed and converted to FASTQ files with Illumina® bcl2fastq (version 2.20.0.422) with predominantly default settings (including adapter trimming). The -no-lane-splitting flag was also added as lanes were not used in this protocol. Alignment and variant calling steps were run as part of the validated bcbio-nextgen cancer somatic variant calling pipeline (version 1.1.3a) (https://github.com/bcbio/bcbio-nextgen). The bcbio-nextgen template that was used can be seen in Supplementary Table 7. The versions of all programs used by bcbio-nextgen are additionally shown in Supplementary Table 8. Briefly, the raw tumor and blood FASTQ files were first processed by Atropos (version 1.1.21) (Didion* et al.* 2017) to clip homopolymers (minimum 8 bases) from the ends of reads. Trimmed FASTQ files were aligned to the rat genome (version rn6) with BWA-mem (version 0.7.17) (Li 2013) with predominantly default settings. The two exceptions were that the -M flag was enabled to mark shorter split read hits as a secondary alignment and seeds with >250 occurrences were skipped (reduced from the default 500).

#### WGS variant calling

Germline variants were called by Varscan (version 2.4.3) (Koboldt* et al.* 2012), GATK HaplotypeCaller (GATK version 4.1.2.0) (McKenna* et al.* 2010, DePristo* et al.* 2011) and Strelka2 (version 2.9.10) (Kim* et al.* 2018) callers. Somatic variants were called by the Varscan (version 2.4.3), Mutect2 (GATK version 4.1.2.0) (Cibulskis* et al.* 2013) and Strelka2 (version 2.9.10) variant callers. In both instances, a variant was accepted as true if it was detected by two of the three callers. Variant allele frequency was calculated from VCFs produced from bcftools call of samtools mpileup. All variants were inspected manually using the Integrative Genomics Viewer (IGV) (Robinson* et al.* 2011) and somatic variants with some germline presence in difficult-to-sequence regions of the genome were removed. The effect of somatic variants was annotated by ensembl VEP (version 95.3). Mutation signatures were generated using MutationalPatterns (Blokzijl* et al.* 2018). Signatures used were single base substitution (SBS) signatures from COSMIC v3 (Alexandrov* et al.* 2019, Tate* et al.* 2019).

#### WGS copy number and structural variant calling

Somatic copy-number profiles were generated by running FACETS on the BCBio aligned BAM files with a rat-specific GC profile (calculated from the rn6 reference genome) and a cval of 1000. SVs were called separately with GRIDSS (version 2.0.0) (Cameron* et al.* 2017). The bam files used by GRIDSS were generated separately to those used for variant calling and CNV detection. Specifically, alignment was performed with BWA-mem (version 0.7.17) with default settings and duplicate marking was performed with samtools (version 1.9). Structural variants were annotated with the StructuralVariantAnnotation R package (https://github.com/PapenfussLab/StructuralVariantAnnotation).

### Custom R analyses

All R analyses were performed with R (version 3.6.0) (https://www.r-project.org/). Circos plots were generated from GRIDSS and FACETS outputs using circlize (Gu *et al.* 2014).

### Comparison of syntenic regions between human and rat chromosomes

A table of human-rat gene homology (hg38 vs rn6) was downloaded from ensembl biomart (ensembl release 96) (Zerbino* et al.* 2018). The database was then filtered down to the rat chromosomes altered in the RS0 model (8, 5, 9 and 14p). Rat cytoband data (rn6) were downloaded from the UCSC annotation database (Raney* et al.* 2014). The proportion of rat-human syntenic genes on each chromosome arm was then calculated by dividing the number of high confidence syntenic genes between arms by the total number of genes on the corresponding human chromosome. GISTIC (version 2.0.23) (Mermel* et al.* 2011) was run on copy-number variation (CNV) profiles of *SDHB* tumors obtained from the TCGA and Castro-Vega papers (Castro-Vega* et al.* 2015, Fishbein* et al.* 2017). The proportion of rat-human syntenic genes was then compared with the frequency of chromosome arm loss (defined as >50% of a chromosome arm) as reported by GISTIC.

### Transcriptional profile

RNA sequencing was performed with an Illumina HiSeq 2000 at the Tufts genomics core facility. Paired-end RNA sequencing data were preprocessed using Trimmomatic (Bolger* et al.* 2014) to filter poor quality reads, then aligned using TopHat2 pipeline (Kim* et al.* 2013). FeatureCounts software (Liao* et al.* 2014) was used to map reads to genes, and edgeR (Robinson* et al.* 2010) was used to calculate RPKM (reads per kilobase of transcript per million mapped reads) (Powers* et al.* 2018).

### Consensus clustering analysis

Rat genes were first mapped to human genes using the biomaRt R package (Durinck* et al.* 2005). The rat RNAseq data were then normalized using the same procedure that was applied to the human samples in the published TCGA analysis (Fishbein* et al.* 2017): rsem normalization, log2 transform and then gene median centered. Genes with 0 reads across all rat samples were removed. The top 3000 most-variable genes by median absolute deviation were selected for cluster discovery using ConsensusClusterPlus as was done for the human clustering (Wilkerson & Hayes 2010, Fishbein* et al.* 2017). We then mapped 1699 rat genes to these 3000 human genes and used these 1699 genes to do a cross-species consensus clustering for 173 human samples and 3 rat samples.

### Metabolomics

Metabolomic analysis was performed as previously described (Powers* et al.* 2018). Briefly, unbiased metabolite profiling was performed using ^1^H NMR, and ^13^C-NMR was used to trace the metabolic fates of glucose and the contributions of glycolysis or anaplerotic TCA cycle pathways to the metabolite profile (Bruntz* et al.* 2017). Replicate s.c. xenograft nodules (each 1 cm in greatest dimension) were analyzed for RS0, RS1/2 and PC12. Pooled tissue from normal rat adrenal medullas was used as an additional SDH-intact control for ^1^H NMR profiling.

### Statistics

Data are presented as mean ± s.e.m. with individual values displayed on graphs. Statistical analysis was carried out using GraphPad Prism software (GraphPad Software Inc). Significance was tested using unpaired Student’s *t*-test, two tailed distribution with *P* < 0.05 set as significant.

### Study approval

All animal procedures performed in these studies were approved by the Institutional Animal Use and Care Committee of Tufts University and Tufts Medical Center.

### Genomics data availability

All WGS and RNA-seq data are available through NCBI short-read archive (SRA) (bioproject ID: PRJNA601534).

## Results

**Figure 1:**
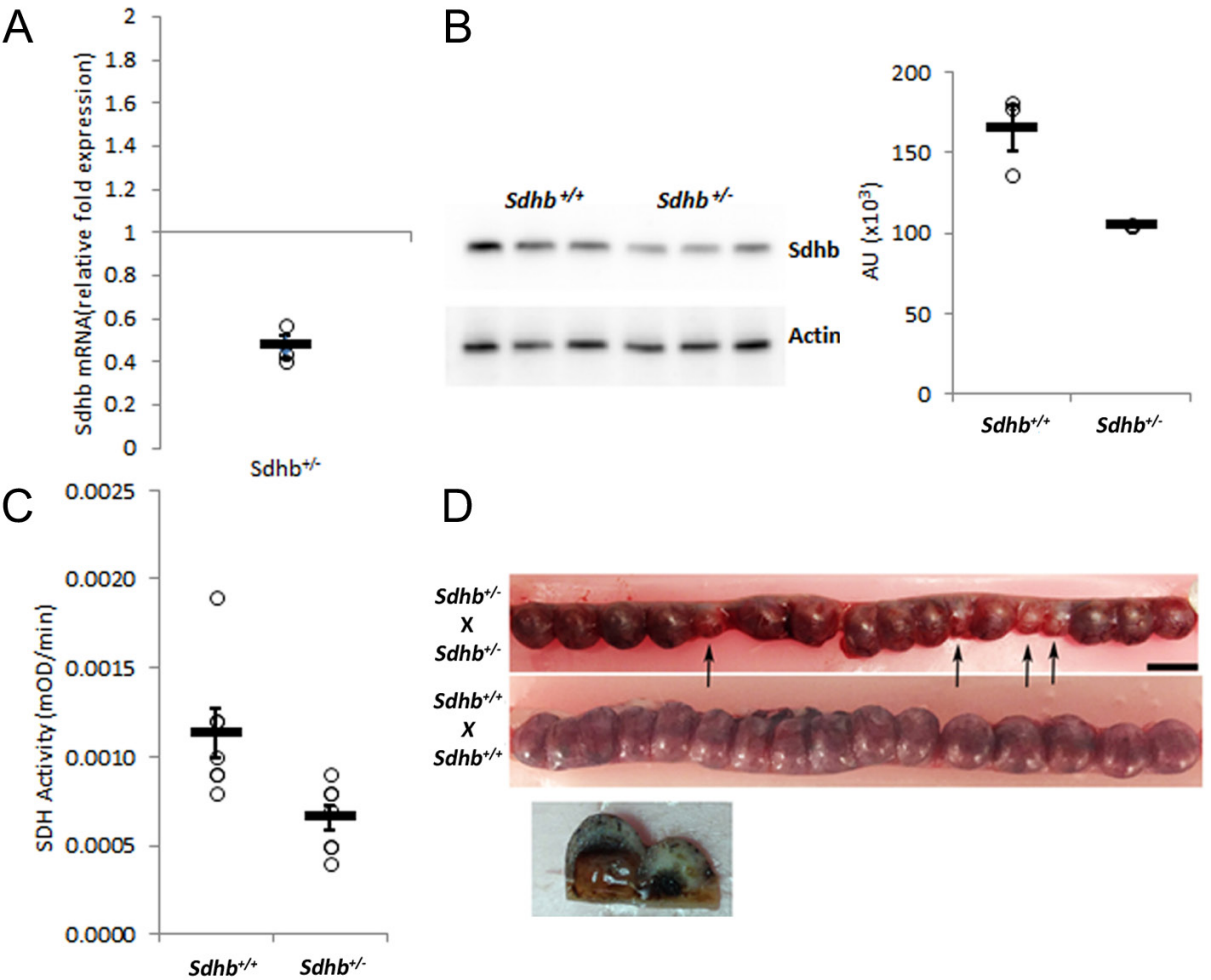
Molecular and functional consequences of heterozygous *Sdhb* deletion. (A, B and C) Comparisons of liver tissue from 2-week-old heterozygous (*Sdhb^+/-^*) and WT (*Sdhb^+/+^*) rats. (A) Decreased Sdhb mRNA (*n* = 3 animals for each group, each point done in triplicate *P < 0.05). (B) Protein expression with densitometric analysis *n* = 3 *P < 0.05; and (C) decreased Sdh enzyme activity (*n* = 7 samples for each group, individual points done in duplicate) *P < 0.05. (D) Opened uteri from 12-day-old gravid rats showing stalled embryos in a representative heterozygous cross (top) compared to WT (bottom). Nine of 31 embryos examined were stalled in *Sdhb^+/-^*x *Sdhb^+/-^* vs 1 of 31 in in WT pregnancies (*P* = 0.0125).

Liver tissue from heterozygous rats carrying each of the three germline *Sdhb* deletions showed approximately 50% reduced expression of Sdhb mRNA and enzyme activity compared to age-matched WT rats and 68% of normal protein expression based on densitometry data. The biological significance of haploinsufficiency was confirmed by crossing *Sdhb* ^+/-^ X *Sdhb* ^+/-^ rats to generate homozygotes, which resulted in embryos developmentally stalled at ~5–6 days, as reported for *Sdhb* ^-/-^ mice (Piruat* et al.* 2004). Approximately 25% of 32 embryos were affected, consistent with the percentage of homozygotes predicted by Mendelian genetics (Fig. 1). In contrast, only one embryo of 31 was stalled in control specimens from WT rats. Because of the apparent functional equivalence of all three *Sdhb* deletions, we arbitrarilly decided to focus on the largest (i.e. 13 base) deletion for colony expansion.

The mean lifespan of rats harboring the 13-base deletion was 88.5 ± 3.0 weeks (range 67–105, *n* = 15) for the non-irradiated vs 69 ± 4 weeks (range 38–104, *n* = 20) for the irradiated group (*P* = 0.0001) and the mean lifespan of 130 weeks for normal Sprague–Dawley rats according to the supplier, Taconic Laboratories (Rensselaer, NY, USA). Macroscopic PCs ~0.3–0.6 cm in greatest dimension were present in three irradiated rats and one non-irradiated. In addition, one irradiated rat developed a carotid body PG. Multiple microscopic adrenal medullary lesions that were cytologically identical to PCs but did not invade or compress the adrenal cortex were present in three irradiated and four non-irradiated animals. Although similar microscopic lesions would be classified as hyperplastic nodules in veterinary pathology literature (Tischler* et al.* 2014), they are classified here as micro PCs in light of current understanding based on molecular studies of small adrenal medullary nodules in humans (Korpershoek* et al.* 2014). Multiple pituitary adenomas were present in both groups of rats, as shown in Supplementary Table 3.

**Figure 2:**
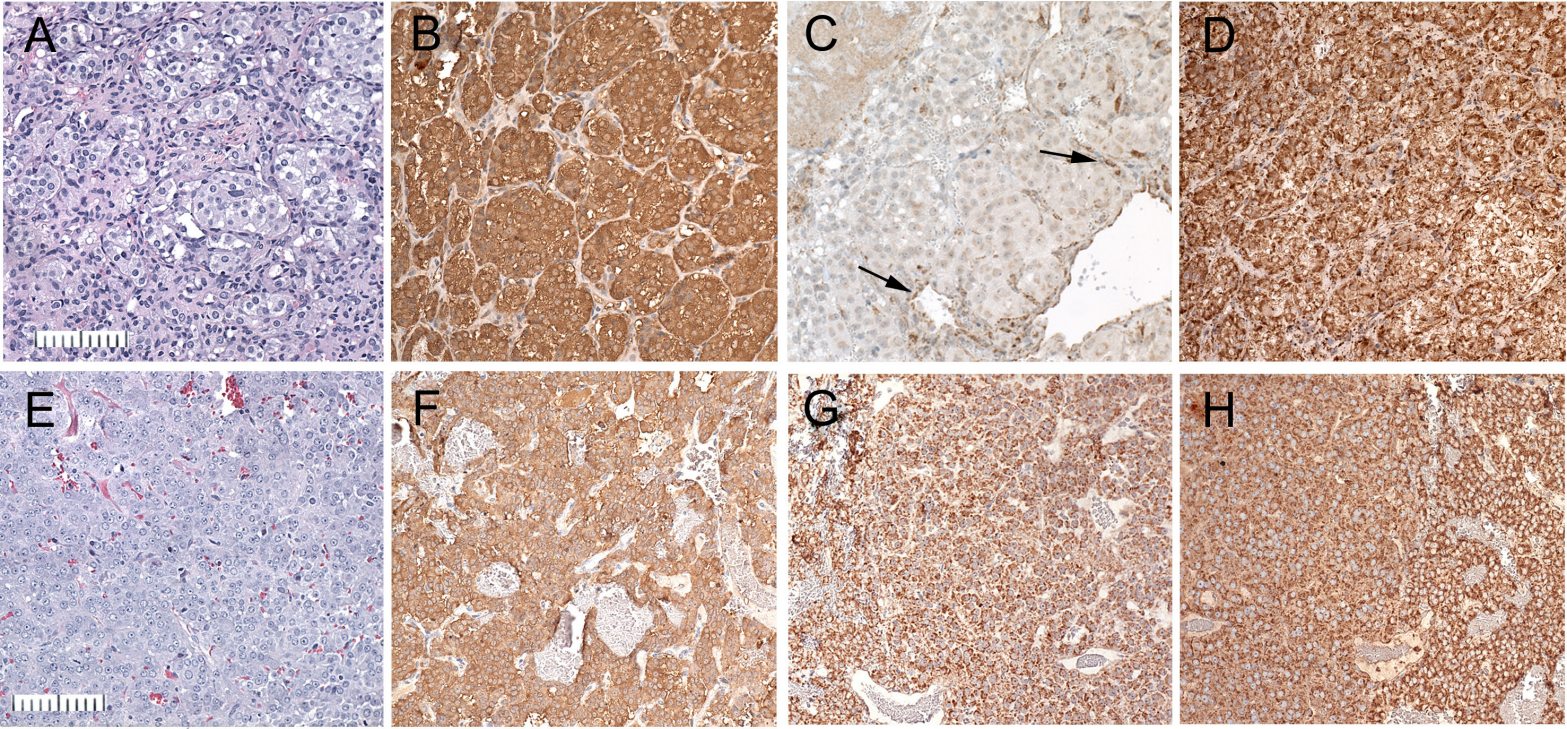
Histology and immunohistochemistry of pheochromocytoma xenografts RS0 (A, B, C and D) and RS1/2 (E, F, G and H). (A and E) Hematoxylin and eosin stain (B and F). Tyrosine hydroxylase RS0 shows classic ‘Zellballen’ pattern characteristic of pheochromocytomas and extra-adrenal paragangliomas, while RS1/2 shows diffuse growth. (C and G) SDHB. RS0 shows loss of granular cytoplasmic staining for Sdhb in tumor cells and retained dark granular staining in endothelial cells (arrows). In contrast RS1/2 shows positive staining in both tumor and endothelial cells. (D and H) SDHA. Both RS0 and RS1/2 show positive staining in tumor cells and endothelilium. Bar = 100 µm.

**Figure 3:**
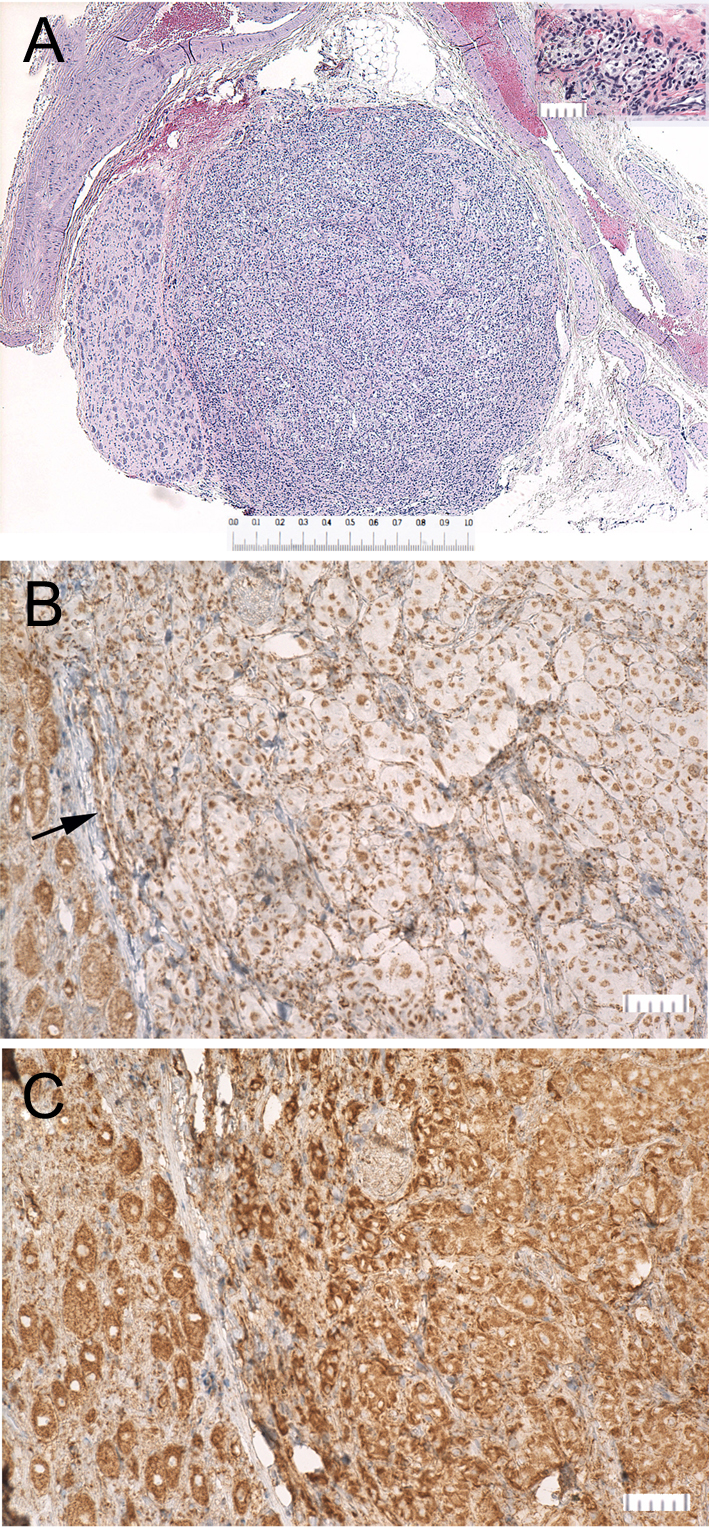
Carotid body paraganglioma. (A) Carotid bifurcation containing a paraganglioma (center) and adjacent superior cervical ganglion (left); Bar = 1 mm. Inset shows the entire normal carotid body from an 18-month-old *Sdhb^+/-^* rat (Bar = 50 um). (B) Immunohistochemical stain for SDHB showing loss of granular cytoplasmic staining in tumor cells and retained dark granular staining in endothelial cells (arrow) and SCG neurons. (C) Tumor cells, neurons and endothelial cells show granular staining for SDHA.

Two distinct, serially transplantable, PC xenograft models, which we have designated as RS0 and RS1/2, were derived at 84 weeks and 74 weeks, respectively, from macro PCs that arose in irradiated rats. RS0 xenografts histologically show sharply defined ‘Zellballen’ architecture, slightly clear cells and prominent blood vessels closely resembling human paragangliomas (Tischler & deKrijger 2015), while RS1/2 shows more diffuse growth (Fig. 2). As an initial screen for Sdh deficiency, both xenograft models and all primary tumors were stained immunohistochemically for SDHB protein (Dahia* et al.* 2005, van Nederveen* et al.* 2009). Only RS0 and the carotid body PG, which was too small to xenograft, were Sdhb-negative, as defined by loss of the granular cytoplasmic staining characteristic of intact SDHB (Figs 2C and 3B). Both were positive for SDHA, which is known to persist in SDH-deficient tumors caused by *SDHB* mutations (Korpershoek* et al.* 2011, Papathomas* et al.* 2015, Bezawork-Geleta* et al.* 2018). Additional immunohistochemical stains demonstrated diffuse immunoreactivity for tyrosine hydroxylase in both RS0 and RS1/2. All pituitary adenomas showed intact staining for both SDHA and SDHB.

**Figure 4:**
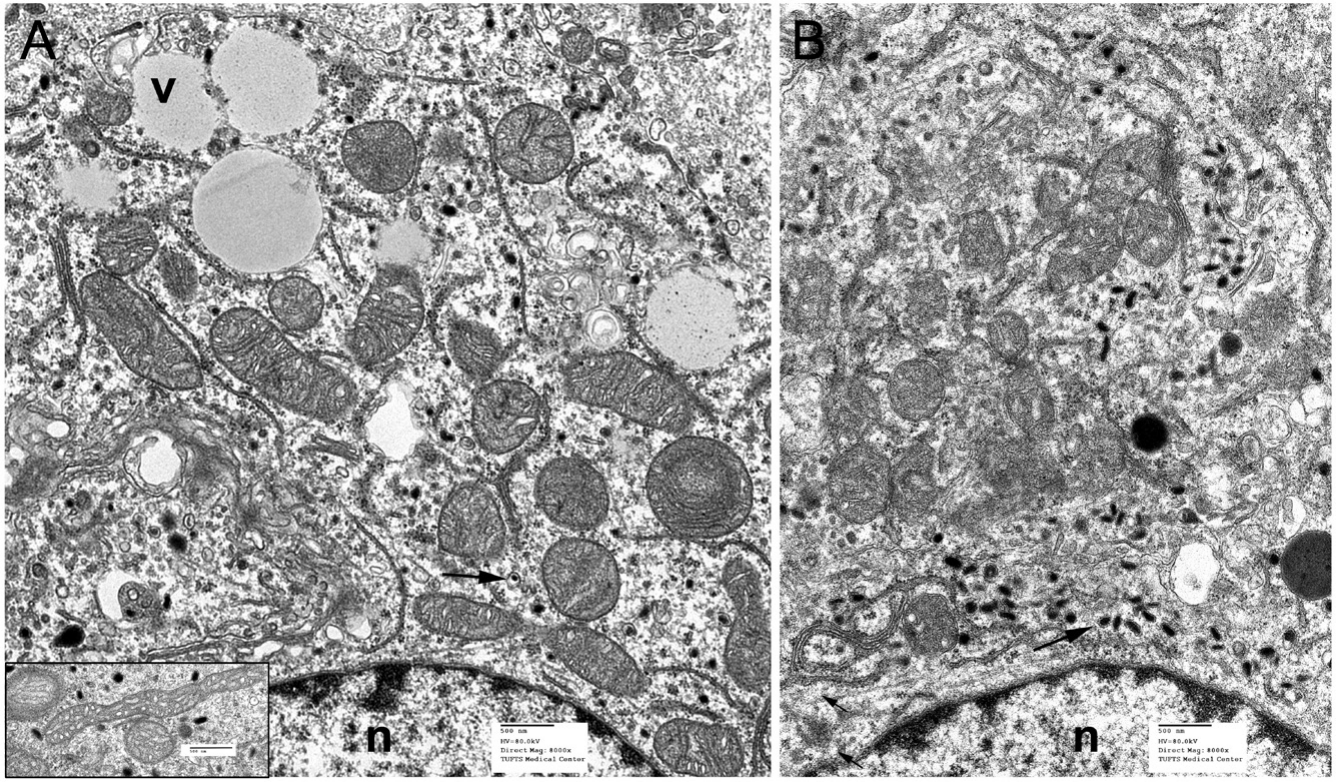
Electron micrographs of RS0 (A) and RS1/2 xenografts (B). Arrows indicate neuroendocrine secretory granules; v, cytoplasmic vacuoles; n, nuclei. Characteristic features of RS0 are cytoplasmic vacuoles and sparse, often tiny secretory granules. In contrast, RS1/2, which is not SDH-deficient, has larger secretory granules and lacks cytoplasmic vacuoles. Inset at left shows elongated mitochondria with tubular cristae that were seen in some RS0 cells.

The most distinctive ultrastructural features of RS0 were relatively sparse secretory granules and cytoplasmic vacuoles similar to those in other SDH-deficient tumors (Powers* et al.* 2018). RS1/2 showed larger and more numerous secretory granules than RS0 and no cytoplasmic vacuoles (Fig. 4). An additional difference was that the mitochondria in some RS0 cells showed prominent tubular or branched cristae and were sometimes also elongated (Fig. 4 inset). These reactive changes have been observed in some SDH-deficient human tumors (Szarek* et al.* 2015) and can result from altered redox balance (Cogliati* et al.* 2016). However, the marked mitochondrial swelling and degeneration reported in the human tumor specimens and some other models (Douwes Dekker* et al.* 2003, Szarek* et al.* 2015, D’Antongiovanni* et al.* 2017, Powers* et al.* 2018) were not seen. The relatively mild mitochondrial changes in RS0 cells are consistent with a recent study indicating that immortalized Sdh-null cells derived from rodent chromaffin cells may be less bioenergetically compromised than Sdh-compromised cells of other types (Kluckova* et al.* 2020).

### Cell culture and establishment of cell lines

**Figure 5:**
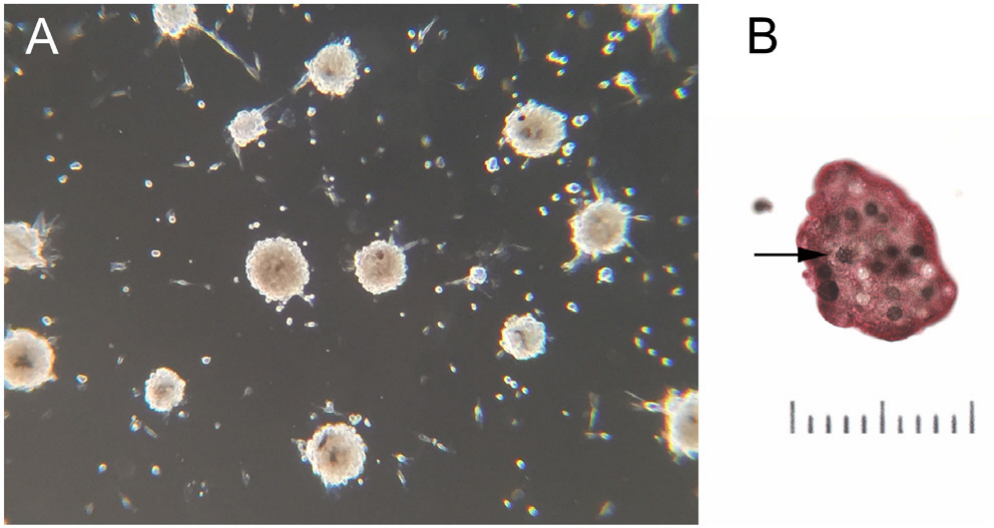
Cell cultures of RS0 cells grown in serum free medium. (A) Darkfield photomicrograph showing typical appearance of tumor cell spheroids, which often form on top of cells attached to the culture dish, then detach and grow suspended in the medium. (B) Cytocentrifuge preparation showing a representative microsphere immunohistochemically stained for tyrosine hydroxylase (red cytoplasm) and bromodeoxyuridine (BrdU, black nuclei) after 7 days of BrdU labeling. White circles within the microsphere are unlabeled nuclei. Arrow indicates a labeled mitotic figure, indicating that incorporating BrdU can go on to divide. Bar = 100 µm.

Preliminary studies showed that cells derived from neither xenograft model would produce a cell line in an atmosphere of 95% air/5% CO_2_ and routine RPMI culture medium with 10% horse serum/5% fetal bovine serum_._ Under those conditions, RS0 cells rapidly accumulated large cytoplasmic vacuoles and died over a period of approximately 2 weeks. In contrast, RS1/2 cells survived for months and did not form vacuoles but slowly dwindled. Optimal cell survival was obtained in 5% O_2_, consistent with the beneficial effect of low O_2_ previously observed with SDH-deficient GIST (Powers* et al.* 2018). However, this was still insufficient to develop cell lines. We, therefore, next tested combinations of 5% O_2_ together with lowered-to-absent serum in medium with stem cell-promoting supplements (Persson* et al.* 2017). Under those conditions, RS0 cells proliferate as a continuous cell line in uncoated plastic culture dishes in serum-free medium as free-floating spheres with a doubling time of approximately 14 days (Fig. 5). A similar-appearing cell line growing in 5% O_2_ was also derived from RS1/2 cells. However, the RS1/2 line requires serum in a low concentration (1% HS/0.5% FBS). For both cell types, preliminary studies showed the use of RPMI 1640, as the base medium is slightly preferable or equivalent to the DMEM/F12 mixture widely used in stem cell cultures.

### Whole genome sequencing

**Figure 6:**
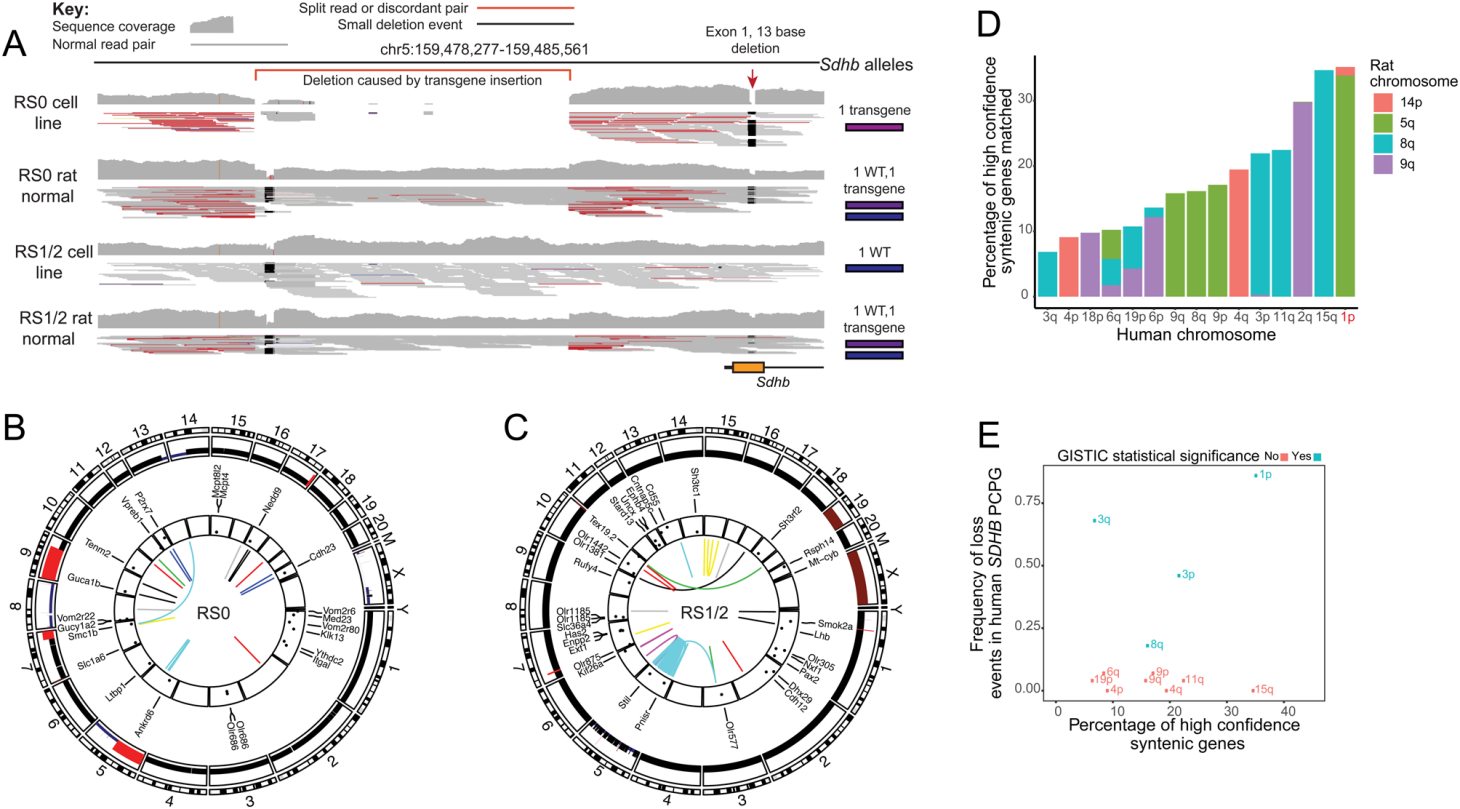
WGS of RS0 and RS1/2 tumor cell lines and their matched germline DNA. (A) Genome viewer snapshot of sequence coverage spanning exon 1 of *Sdhb* and the genomic region immediately 5′ of *Sdhb* (~1.4 kb in total). A 13-bp deletion within exon 1 of *Sdhb* was detected in the germline DNA of both RS0 and RS1/2. The exon 1 deletion was present in all sequencing reads in tumor cell line RS0, indicating that the WT *Sdhb* allele had been lost during tumorigenesis. Conversely, the TALEN-edited *Sdhb* allele was lost during tumorigenesis of tumour cell line RS1/2. In addition to the exon 1 deletion, a larger 729-bp deletion was also detected upstream of *Sdhb,* indicated by the presence of discordant or split reads spanning the deletion breakpoint (these reads are highlighted in red). The deletion upstream of *Sdhb* is also supported by a precipitous drop in sequence coverage spanning the 729-bp deletion. Like the 13-bp exon 1 deletion, the 729-bp deletion was detectable in the germline DNA of RS0 and RS1/2, indicating that it was introduced by TALEN gene-editing. (B) A circos plot describing the somatic alterations that occurred in the formation of RS0. From outside to inside, tracks are as follows: rat cytoband, total copy-number variation (CNV) as called by FACETS (black signifies *n* = 2, red signifies *n* > 2 and blue signifies *n* < 2), names of genes called by BCBio ensemble variant calling as having a coding mutation, the allele frequency of these mutations and the innermost track represents the structural variants called by GRIDSS. (C) A circos plot describing the somatic alterations that occurred in the formation of RS1/2. Tracks are in the same order as described in (B). (D) The percentage of high confidence orthologous genes matched between rat and human comparing chromosome arms called by FACETS as being altered in RS0 (either gain or loss). (E) Comparison of high confidence orthologous genes from D (further filtered only to chromosomal loss events in RS0) against the frequency of chromosomal arm level loss events in human *SDHB* PCPG as determined by GISTIC. Human copy-number variation (CNV) data were taken from publicly available copy-number profiles cited in the Materials and methods section. Chromosome arms colored in blue were called as statistically significantly altered by GISTIC (false discovery rate <0.05). Chromosome arm level losses of 1p, 3q, 3p and 8q are frequent and statistically significant events in human PCPG. These regions are overlapping with syntenic genomic regions that have undergone somatic loss in rat model RS0.

We performed WGS to verify genetic loss of WT *Sdhb* and to identify additional somatic changes in RS0 and RS1/2 tumor cells. WGS was initially performed on RS0 xenograft tissue; however, some ambiguities resulted from the presence of mouse DNA sequences introduced by the host animal. In order to obtain cleaner data, WGS was repeated using DNA extracted from RS0 and RS1/2 cultured cell lines devoid of mouse stromal and immune cells. Focused analysis of sequence coverage of *Sdhb* identified the expected 13-bp deletion in exon 1 plus a 729-bp deletion immediately upstream of the *Sdhb* promoter in the RS0 germline DNA (Fig. 6A). As shown in Fig. 6A, there is clear evidence of a 729-bp hemizygous deletion in both the normal germline DNA and tumour DNA of RS0 and RS1/2. The deletion is inferred by the sharp reduction in read depth at the genome co-ordinates upstream of SDHB as indicated and the paired reads that span the deletion, which are marked in red. The deletion was certainly a result of the TALEN process and not radiation. Inference of read coverage, and genome-wide copy-number analysis for cell line RS0 showed somatic segmental loss of chr5 including the WT *Sdhb* allele (Fig. 6B). In cell line RS1/2, we observed a chromothripsis involving chr5 resulting in loss of the TALEN-edited non-functional *Sdhb* allele but retention of the *Sdhb* WT allele (Fig. 6C). These data are in keeping with the negative SDHB protein expression in RS0 but positive SDHB staining in RS1/2.

Both RS0 and RS1/2 cell lines had low mutation burden (RS0, 0.8 mutations/Mb; RS1/2 0.96 mutations/Mb). The highest proportion of base substitutions involved C>T transitions, but no dominant mutational signatures were observed in either cell line (Supplementary Fig. 2). With respect to mutations and structural alterations, no somatic lesions in gene orthologues previously found to be mutated in human PCPG were identified (Supplementary Tables 4 and 5). A ~108-kb homozygous deletion involving exon 1 of *Cdkna* and complete loss of *Cdkn2b* was identified in cell line RS0. Inspection of the WGS sequence data in the matched xenograft, however, showed no evidence of this deletion event in the original RS0 tumor, therefore it was unlikely to be a founding event in RS0 tumor cells (Supplementary Fig. 3). In RS1/2, two mutations of unknown significance were detected in known cancer genes (*Stil* and *Ext1*). Furthermore, numerous structural alterations were identified in cell line RS1/2 involving chromothripsis of chromosome 5. Breakpoints were detected within cancer genes *Arid1a Nfib and Psip1*, the latter involving a gene fusion with *Zbtb48*. The principal driver gene mutation in RS1/2 is not apparent but may involve haploinsufficiency of one or more of these cancer genes or other genes on chr5. Interestingly, in cell line RS1/2 we observed a missense mutation at 10% variant allele frequency in cytochrome B (Mt-cyb). Despite the paucity of pathogenic mutations in both cell lines, we observed large segmental copy-number loss events in RS0 that are syntenic with frequently altered chromosomal regions in human *SDHB*-associated PCPG tumors (Fig. 6D and E). These events included syntenic loss of human chr1p, chr2q, chr3, chr8q and chr18p. We reasoned that these structural events are likely to be important in co-operating with *SDHB* loss of function to promote tumorigenesis, and provide further evidence that RS0 has the genomic hallmarks of human *SDHB* pheochromocytoma.

### Metabolite profile

The presence of SDH deficiency was confirmed by high levels of succinate accumulation (Table 1). In RS0 xenografts, succinate was the third most abundant metabolite, in contrast to both RS1/2 and adrenal medulla. Lactate was the most abundant metabolite measured, consistent with increased production of lactate and induction of lactate dehydrogenase in SDH-deficient tumors and cell lines (Lussey-Lepoutre* et al.* 2015). *In vivo* ^13^C-glucose labeling showed robust incorporation of glucose into lactate and succinate and lesser incorporation into alanine and glutamate. The labeled glutamate contained almost the same amounts of (^13^C2-4,5)Glu and (^13^C2-2,3)Glu isoptomers, indicating approximately equal activity of pyruvate dehydrogenase (PDH) and pyruvate carboxylase (PC), respectively (Lussey-Lepoutre* et al.* 2015, Bruntz* et al.* 2017). This result is consistent with previous studies showing increased utilization of the anaplerotic pathway catalyzed by PC in Sdh-deficient mouse cell lines (Lussey-Lepoutre* et al.* 2015).

**Table 1:** NMR metabolomic profiles of RS0, RS1/2 xenografts and rat adrenal medulla (RAM).

RAM (20 pooled)		RS0 (*n* = 4)		RS1/2 (*n* = 3)	
			Mean ± s.e.m.		Mean ± s.e.m.
The ten most abundant detectable metabolites (µmol/mg of tissue)					
Epinephrine	16.15	Lactate	15.18 ± 3.66	Norepinephrine	33.87 ± 29.41
Norepinephrine	12.33	Taurine	12.11 ± 2.69	Lactate	11.45 ± 2.18
Glucose	2.31	**Succinate**	5.99 ± 1.19	Taurine	10.38 ± 0.83
Lactate	0.89	Glycine	3.47 ± 0.75	Ascorbate	7.611 ± .42
Taurine	0.61	Glutamate	2.57 ± 0.58	myo-Inositol	6.33 ± 2.80
ATP (or ADP)	0.59	Ascorbate	2.57 ± 0.64	Glutamate	4.16 ± 0.97
Ascorbate	0.44	Alanine	2.08 ± 0.45	Dopamine	2.55 ± 2.04
AMP	0.30	Creatine	1.39 ± 0.28	O-Phosphoethanolamine	2.06 ± 0.25
ADP (or ATP)	0.21	sn-Glycero-3-phosphocholine	1.29 ± 0.34	AMP	1.85 ± 1.39
Glutamate	0.17	myo-Inositol	1.21 ± 0.26	Betaine	1.83 ± 0.31
**Succinate**	0.01			**Succinat**e	0.29 ± 0.10
30 of 67				30 of 67	
Catecholamine and metabolite profile (µmol per mg of tissue)					
Dopamine	0.07		0.38 ± 0.08		2.55 ± 2.04
3,4-Dihydroxybenzeneacetate^a^	0.00		0.05 ± 0.02		0.23 ± 0.05
Norepinephrine	12.33		0.06 ± 0.03		33.87 ± 29.41
Normetanephrine	0.00		0.02 ± 0.01		0.00 ± 0.00
Epinephrine	16.15		0.00 ± 0.00		0.81 ± 0.78
Tyrosine	0.00		0.11 ± 0.01		0.03 ± 0.00

^a^3,4-Dihydroxyphenylacetic acid (DOPAC).

Similar to SDH-deficient human paragangliomas, the catecholamine profile of RS0 xenografts showed a predominance of dopamine, low levels of norepinephrine and undetectable epinephrine (Table 1).

### Transcriptome and immunoblots

RS0 xenografts show high expression of canonical cluster 1 markers associated with the Hif2a regulatory network (Table 2) and with hereditary *SDHB* mutations in the Cancer Genome Atlas (TCGA) (Fishbein* et al.* 2017) and previous comparable studies (Burnichon* et al.* 2011, Castro-Vega* et al.* 2015). These include Hif2a (also known as Epas1) and its targets including Vegfa and Adm (Table 2, and complete list in Supplementary Table 6). RNAseq also confirms the near total loss of Sdhb mRNA in RS0 xenografts, with residual low level expression consistent with the presence of blood vessels and other non-neoplastic cells (Fig. 2C). A novel finding is the presence of concomitant, though lesser, reduction in mRNA transcripts of other SDH subunits (Supplementary Table 6). Surprisingly, RS1/2 also shows modest reductions in multiple SDH subunits despite the presence of immunoreactive SDHB and a relatively low level of Hif2a (Supplementary Table 6).

**Table 2:** RNAseq data comparing expression of Epas1 and genes in its regulatory network in RS0 and RS1/2 xenografts and pooled rat adrenal medullas (RAM).

Gene^a^	RAM (RPKM^b^)	RS0 (RPKM)	RS1/2 (RPKM)	RS0/RAM	RS1/2/RAM
**Epas1**	80.01	**1074.63**	76.62	**13.43**	0.96
Egln3	1.44	23.29	0	16.17	0
Bhlhe40	10.99	114.0	11.77	10.37	1.07
**Vegfa**	51.43	**607.36**	62.04	**11.81**	1.21
Twist1	1.86	14.82	0.50	7.95	0.27
Serpine1	2.65	241.62	5.9	91.18	2.23
Adora2a	5.41	593.88	2.99	109.78	0.55

^a^Genes listed are from the NCBI HIF-2-alpha transcription Network (Biosystems NCBI, https://www.ncbi.nlm.nih.gov/biosystems/137956?Sel=geneid:2034#show=genes). Those with the highest in the ratios in RS0 to RAM are shown. ^b^Raw data are expressed as reads per kilobase of transcript per million mapped reads (RPKM). The constitutive expression of Epas1 in RAM is consistent with findings in normal embryos (Favier* et al.* 1999).

**Figure 7:**
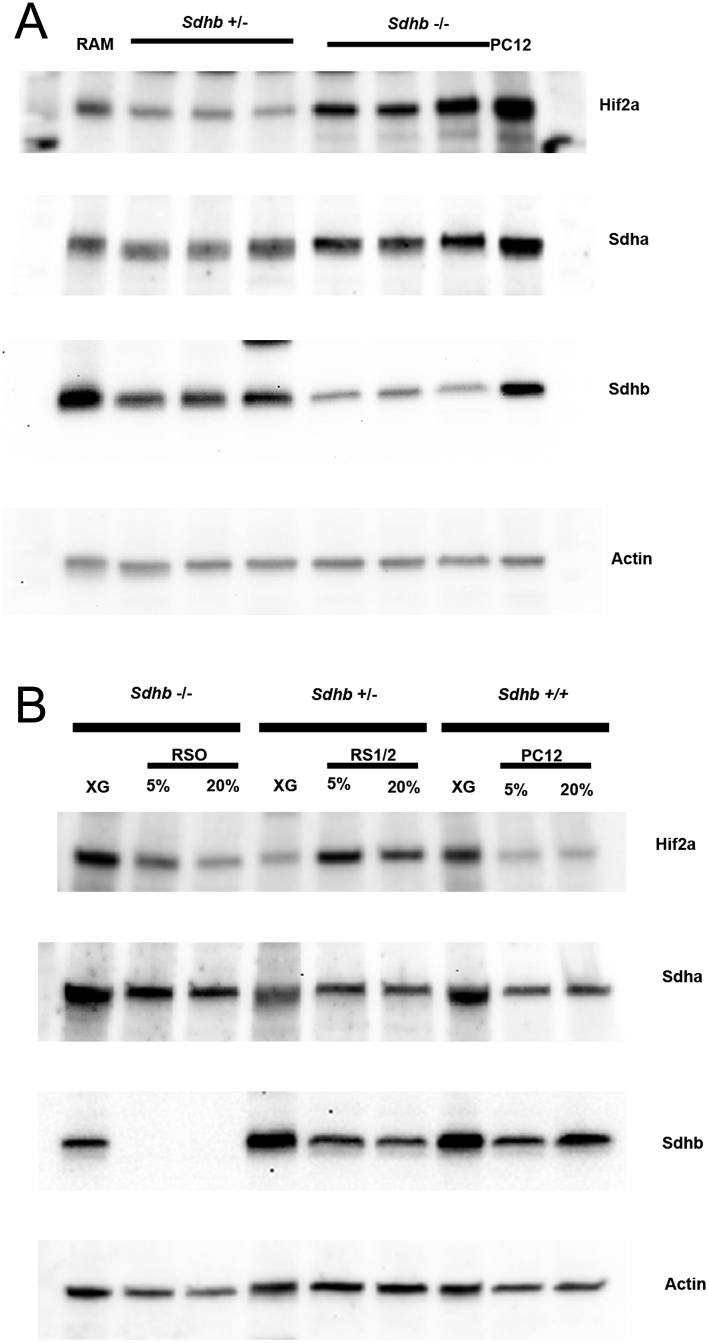
Immunoblots for HIF2A, SDHB and SDHA. (A) Representative immunoblot comparing levels of HIF2A, SDHB and SDHA in RS0 and RS1/2 xenografts. A single immunoblot from a 4–15% gradient polyacrylamide gel was consecutively probed, stripped and re-probed for each of the indicated proteins. RAM (rat adrenal medulla) and PC12 (xenograft produced in NSG mouse from passage 40 cells) were run as controls. (B) Representative immunoblot comparing RS0, RS1/2 and PC12 xenograft tissue (XG) to corresponding cell lines in culture maintained in either 5% or 20% O_2_. Single immunoblot stripped and reprobed as in panel A.

To further test how expression of the critical markers HIF2A and SDHB at the protein level correlates with immunohistochemical and RNAseq data, immunoblots of RS0 and RS1/2 xenografts were probed for these proteins in parallel with their corresponding cell lines. The PC12 rat pheochromocytoma, which is known to express HIF2A (Bechmann* et al.* 2019) but be *Sdhb*- intact, served as a positive control. In xenograft tissue, RS0 shows high HIF2A expression consistent with its high expression at the RNA level, while expression in RS1/2 is relatively low. Interestingly, in cell cultures this pattern is reversed as a result of increased expression in RS1/2. Cell cultures also show that protein expression is not completely unresponsive to O_2_ concentration in either RS0 or RS1/2 cells. Importantly, however, expression in RS0 cells at 5% O_2_, which approximates the concentration in solid tumors (Carreau* et al.* 2011), is comparable to expression in xenograft tumor tissue (Fig. 7). Immunoblots confirm the loss of SDHB in RS0 cells, while SDHA is both retained and slightly increased in primary Sdhb^-/-^ vs Sdhb^+/-^ xenografts (Fig. 7A) and in RS0 compared to RS1/2 cells (Fig. 7B). This increase may be related to a reported role of Sdha in an alternate assembly of respiratory complex 2 that decreases metabolite synthesis and cell proliferation in response to knockdown of SDHB (Bezawork-Geleta* et al.* 2018).

### Cross-species clustering analysis

In the TCGA study, consensus clustering divided 173 human samples of PC/PGs tumors into four molecularly defined groups: a kinase signaling subtype, a pseudohypoxia subtype, a *WNT*-altered subtype and a cortical admixture subtype (Fishbein* et al.* 2017). We performed cross-species consensus clustering analysis to test where among those subtypes the rat tumor samples would correspond. To do this, the rat RNAseq data were first analyzed to identify human homologs among the 3000 most highly expressed SDHB-associated genes used in the TCGA study. This yielded a set of 1699 overlapping genes. Consensus clustering was performed as described in Methods. First, using the 1699 gene sample set to re-analyze the TCGA data, the vast majority of the human TCGA data sets clustered to the same group as originally described for the 3000 genes in the published TCGA study, indicating that the reduced consensus sample set does not adversely affect the clustering results. When all three rat samples were then clustered with the human samples, the rat samples were all clustered together, indicating that the rat samples are more similar to each other than to the human samples (Supplementary Fig. 4A). To avoid this complication, each rat sample was clustered individually with the human samples in order to minimize the species effects. When this was done, all three rat tissue samples, rat adrenal medulla, RS0 and RS1/2, were clustered with the human pseudohypoxic cluster (Supplementary Fig. 4B, C and D).

## Discussion

We have developed two serially transplantable xenograft and cell line PC models, which we call RS0 and RS1/2, from rats with a heterozygous germline deletion in *Sdhb*. RS0, which is Sdh deficient, is genomically comparable to its human counterpart and differs from existing models in that it arose in an animal with a hereditary *Sdhb* mutation. RS 1/2, which was derived from a different primary PC, has lost the mutated *Sdhb* allele retains one WT allele. RS1/2 shows some succinate accumulation consistent with haploinsufficiency but is not fully Sdh deficient. It differs from RS0 in showing intact staining for SDHB, a greater degree of neuroendocrine differentiation and a largely different transcriptome, although some markers are shared. RS1/2 serves both as a control for studies of RS0 and as a potential tool for studying patients’ tumors that may be haploinsufficient for SDH but have undetermined driver mutations. The main focus of this communication is on RS0 and RS1/2 xenografts, which would have the greatest immediate relevance to human tumors *in vivo*. Subsequent publications will entail detailed characterization of the cell lines and their applications.

RS0 and RS1/2 originated and have been maintained in the form of xenografts that were never in cell culture – that is, the equivalent of early passage human PDX models. They may therefore be especially valuable for pre-clinical drug testing. These xenografts grow slowly enough to faithfully represent aggressive human PGs, but rapidly enough to be practical for testing and imaging. Since there is a pending clinical trial of an EPAS1 inhibitor for metastatic PG (NCT02974738) (Tella* et al.* 2017), RS0 xenografts might be immediately relevant for pre-clinical drug testing as well as basic research. Features that RS0 xenografts share with SDH-deficient human PGs include identical histology (Tischler & deKrijger 2015), loss of SDHB protein with retention of SDHA (Korpershoek* et al.* 2011, Papathomas* et al.* 2015) and expression of the neuroendocrine markers TH and chromogranin A (Letouze* et al.* 2013). The presence of these markers indicates that they are not an irrelevant or dedifferentiated cell type, while the relatively low expression is comparable to that in human tumors (Letouze* et al.* 2013). Shared molecular markers include accumulation of a high concentration of succinate, metabolic rewiring and a transcriptional profile including high expression of multiple markers directly sensitive to impaired electron transport or hypoxia. The latter include Hif2a and its downstream targets including Vegfa.

Several highly expressed genes in RS0 xenografts are strong direct indicators of adaptive responses to the pathobiology of SDH deficiency. These include *Cox4i2* (cytochrome c oxidase subunit 4I2), which catalyzes electron transfer from reduced cytochrome c to oxygen, and *Ndufa4l2* (mitochondrial complex associated like 2), which encodes a subunit of Complex I of the respiratory chain. These markers are two of the three ‘exemplar genes’ cited in the TCGA study for being consistently associated with pseudohypoxia in human PGs (Fishbein* et al.* 2017) and Ndufa4/2 is of particular interest in that complex 1 activity is reportedly upregulated in *SDHB* mutated human PGs (Pang* et al.* 2018). However, the third exemplar gene, *Ntng2* (netrin-G2) which modulates development of neuronal circuits and is less obviously related to SDH deficiency, is also not highly expressed in RS0. This is the case for many, but not all, other apparent mismatches. Notably, the hypoxia-inducible factor *Egln3* (egl-9 family hypoxia-inducible factor 3, also known as Phd3), which is highly expressed in RS0, has been strongly associated with pseudohypoxia in *VHL* but not *SDHB*- mutated human tumors (Burnichon* et al.* 2011). While the transcriptome of RS0 is not a perfect match to human cluster 1, many of the mismatches that are peripherally related to the primary functional defects might reflect different species, different anatomic sites (Fliedner* et al.* 2018) and different cells of origin within an anatomic site or different developmental stages in which the human vs rat tumors originate. A particular finding suggesting an influence of developmental stage is the overexpression of Ret in RS0. Ret is widely expressed in early adrenal development but is then downregulated in maturing chromaffin cells and maintained in neurons (Powers* et al.* 2009).

An important aspect of this study is the use of normal adrenal medullary tissue as a reference. This contrasts with profiling studies of human PCPG, in which the different tumor clusters were compared only to each other and to an unrelated reference DNA (Burnichon* et al.* 2011, Fishbein* et al.* 2017). By specifically focusing on large differences between a tumor and its tissue of origin, rather than solely between subsets of tumors, it may be possible to find new drug targets in the HIF2A transcription network that entail fewer systemic side effects than targeting HIF2A itself. Hypothetically, those systemic effects could include depletion of normal stem cells (Hammarlund* et al.* 2018). A disadvantage of normal adrenal medulla is the potential confounding effect of residual adrenal cortex, which is inevitably present, in some analyses. However, by selectively focusing on large differences in specific markers, it is possible to discern distinctive characteristics of the cells of interest, as shown in the TCGA ‘cortical admixture’ cluster. In addition, it may be possible to calculate the proportion of cortical contamination (Fliedner* et al.* 2010). An interesting observation in this study was that carefully dissected normal rat adrenal medulla clustered in the pseudohypoxic group. While this might partly represent an artifact of cross-species clustering, it is consistent with previous reports of HIF2A expression in normal sympathoadrenal development and function (Favier* et al.* 1999, Richter* et al.* 2013).

Although PDX-like xenografts are an excellent model for pre-clinical drug testing (Shultz* et al.* 2014), cell lines are needed for mechanistic studies. Availability of cell lines also makes models accessible to many more researchers than those who would work with xenografts alone. Our cell culture studies were undertaken both to establish cell lines from RS0 and RS1/2 xenografts and to test whether non-conventional culture conditions that we previously demonstrated to be favorable to survival of cells from a unique SDH-deficient human GIST (Powers* et al.* 2018) would apply to these new models. Our results replicated our previous finding that routine ‘normoxic’ culture conditions are deleterious. In addition, we demonstrated an adverse effect of serum, which has been reported by others in PDX-derived cultures of human neuroblastoma. The deleterious effect of oxygen is not surprising because pO_2_ *in vivo* has been measured at ~41 mm Hg in normal liver (equivalent to ~5% O_2_) and ranged from 0–54 mm Hg in tumors of different types (Carreau* et al.* 2011). In both normal and neoplastic cells, low O_2_ may help to maintain stem cells (Hammarlund* et al.* 2018). The beneficial effects that we observed with low or absent serum and stem cell-promoting medium supplements in establishing the RS0 and RS1/2 cell lines is entirely consistent with a recent report by Persson *et al.* that neuroblastoma cells cultured from human patient-derived xenografts differentiate in serum but remain tumorigenic when propagated in stem cell medium (Persson* et al.* 2017). It remains to be determined how these new insights will affect future efforts to develop cell lines of human PC and PG. However, our cell culture findings and those of others suggest a need for radical departure from conventional methods, perhaps especially for SDH-deficient tumors. This applies both to model development and pre-clinical drug testing, which is usually conducted in 95% air/5% CO_2_ and may thereby exaggerate the efficacy of many chemotherapeutic agents (Carreau* et al.* 2011). However, individual tumors and different tumor types (Burgess* et al.* 2017) will likely require individualized culture conditions. It is well known that genetic and epigenetic characteristics of individual cancers and types of cancer can dictate different approaches to treatment (Burgess* et al.* 2017).

Multiple factors likely contributed to the development of these new rat models, including the innate proclivity of aging rats to develop PCs, combined with postnatal irradiation. Both of these have previously been reported for other rodent PC models (Powers* et al.* 2000). Very little is known about the genetic changes underlying the general susceptibility of rats to pheochromocytomas. The most extensively characterized and relevant model is MENX, which is caused by a loss-of-function mutation in *Cdkn1b*, which encodes the cyclin-dependent kinase inhibitor p27Kip1. This change was not found in RS0 or RS1/2, and, to our knowledge, whole genome sequencing has not been performed on MENX rats. Comparisons of these might yield useful information. It is plausible that, in our models, radiation caused somatic genetic changes that accelerated the growth of spontaneous PCs and caused one of the tumors to become SDH deficient. However, consistent with human PCPG, both RS0 and RS1/2 had very low mutation burdens, very few gene coding mutations and relatively stable genomes. Chromothripsis of chr5 caused by a single catastrophic event was likely important for tumorigenesis of RS1/2, and exposure to ionizing radiation may be implicated. However, chromothripsis is also a feature of some primary human PCPG (Flynn* et al.* 2015), which ostensibly arises in the absence of radiation exposure.

As in human PCPG, few co-operative DNA mutations could be found in rat PC and the tumor genome is typified by recurrent chromosomal arm level loss events resulting in presumed haploinsufficiency of multiple genes. Chromosomal loss events syntenic with recurrently altered chromosomal regions in human SDHB-associated PCPG indicate that the RS0 model faithfully represents the genome of human PCPG and therefore may be useful for dissecting therapeutic vulnerabilities based on common co-operative somatic changes. A homozygous deletion was detected in cell line RS0 involving two neighboring tumor suppressor genes *Cdkn2a* and *Cdkn2b*. This event is predicted to cause complete loss of function of the protein p19ARF, which is encoded by an alternative reading frame of *Cdkn2a*. p19ARF is a negative regulator of MDM2 and loss of p19ARF is known to cause indirect suppression of p53 through the ARF-MDM2-p53 tumor-suppressor axis (Eischen* et al.* 1999). However, because the *Cdkn2a* deletion was not detected in the RS0 xenograft, it most likely was not involved in early tumorigenesis of RS0. The existence of the *Cdkn2a* deletion may further increase the relevance of the RS0 cell line as a pre-clinical model because *p16INK4A/Cdkn2a* gene expression is frequently downregulated in human PCPG, either by genetic abnormalities or promoter methylation (Muscarella* et al.* 2008), and is associated with poor prognosis (Kiss* et al.* 2008).

Two hypotheses were implicit in the protocol design and outcome this study. The first is that unpredictable haploinsufficiencies resulting from radiation damage or other causes may account for features required for tumorigenesis in addition to loss of Sdhb, likening the RS0 model to human PCPG. This might explain why previous (’clean’) gene knockouts of SDH subunits in various combinations with targeted loss of selected tumor suppressors, for example, in mice, have failed, because of their inability to achieve these other genetic effects. The second hypothesis is that rats are innately more susceptible than mice to this type of tumor. It is therefore of interest that we unsuccessfully tested a similar protocol in mice prior to undertaking the rat project. The radiation protocol was used initially by Jacks *et al.* to increase the frequency of pheochromocytomas in heterozygous Nf1 knockout mice (Jacks* et al.* 1994) and then in our laboratory to establish the derivative MPC cell lines (Tischler* et al.* 1995, Powers* et al.* 2000). Even more important than radiation, outbreeding of the 129SV*^Nf1^* ^+/-^ mutation carriers to WT C57BL6 mice was essential for tumorigenesis, possibly reflecting different levels of tumor suppressor genes expressed in different mouse strains (Hawes* et al.* 2007). In attempting to develop a Sdhb-null mouse model, we followed exactly the protocol previously used to develop MPC cells. We obtained four male 129SV*^Sdhb^* ^+/-^ mice from Dr Louis Maher at the Mayo Clinic (Maher* et al.* 2011) and outbred them to WT C57BL6 mice. Over the ~2-year lifespan study, pheochromocytomas developed in 3 of 44 irradiated *Sdhb*+/- mice and 1 of 10 in irradiated WT mice. However, all of the tumors were Sdh-intact by immunohistochemistry and none gave rise to cell lines (previously unpublished data). Cumulatively, our experience suggests that the success of the present project resulted from favorable features of rat biology together with irradiation. However, it is possible that a comparable mouse model might still be developed using mice with a different genetic background or by using a larger number of mice.

In summary, we have developed xenograft and cell line models called RS0 and RS1/2 from PCs that arose in rats with a heterozygous germline mutation in *Sdhb*. RS0 closely recapitulates the genotype and phenotype of hereditary *SDHB*-mutated human PCPG and appears to be a promising model for pre-clinical studies of these tumors. In addition, we identified a carotid body paraganglioma that also appeared to be SDH-deficient based on immunohistochemistry. That tumor was too small to graft or culture and it is possible that additional small paragangliomas were missed amid the large amount of fat in aged rats. This new model may be useful both for pre-clinical drug testing and for basic research aimed at understanding mechanisms involved in the development and progression of SDH-deficient human PCPG.

## Supplementary Material

Supplementary Figure 1. The TALEN design for Sdhb disruption. The Sdhb gene is located on rat chromosome 5. Exon 1 was selected as the TALEN target site. TALEN mRNA was generated by in vitro transcription, which was then injected into fertilized eggs for KO rat production. The founders were genotyped by PCR followed by DNA sequencing analysis. The mRNA transcribed from the targeted allele with frameshift undergoes nonsense mediated decay.

Supplementary Figure 2. (A) Mutation signatures of RS0 and RS1/2. The coding region of rat was determined to be 37.48 Mb. RS0 had a mutational load of 30/37.48 = 0.8004269 muts/Mb. RS1/2 had a mutational load of 36/37.48 = 0.96 muts/Mb. Mutation signatures were generated against both models using MutationalPatterns (referenced in Methods). Signatures used are single bases substitution (SBS) signatures from COSMIC v3. The left panel describes the relative contribution of each signature to the overall profile of the sample and the right panel describes the total number of mutations attributed to each signature. While trinucleotide context was obtained from the rat genome (rn6) the signatures were derived from human data and this may have affected the results. (B) The relative contribution to the mutation signatures of each of the 96 possible SBS in each model.

Supplementary Figure 3. Genome browser snapshot of sequence coverage involving breakpoints spanning a ~108,205bp deletion encompassing exon 1 of Cdkn2A in cell line RS0. Top: RS0 xenograft; Middle: RS0 cell line; Bottom: matching normal adrenal. Mate-pair reads spanning breakpoints left (chr5:107827646-107827651) and right (chr5:107935852-107935857) are highlighted in red.

Supplementary Figure 4A. Heatmap of cross-species clustering analyses for rat adrenal medulla (RAM), RS0 xenograft and RS1/2 xenograft. (A) All 3 rat samples. Red arrows indicate the positions of the rat samples (blue), which fall within the human-derived pseudohypoxic cluster (orange). Note a small number of human tumors moved from their TCGA-assigned cluster, which was based on 3000 genes, to other clusters when the TCGA data were reanalyzed with the 1699 gene set used for cross-species clustering. Most of these are cortical admixture to kinase (pink to green).

Supplementary Figure 4B. Heatmap of cross-species clustering analyses for rat adrenal medulla (RAM), RS0 xenograft and RS1/2 xenograft. (B) RAM. Red arrows indicate the positions of the rat samples (blue), which fall within the human-derived pseudohypoxic cluster (orange). Note a small number of human tumors moved from their TCGA-assigned cluster, which was based on 3000 genes, to other clusters when the TCGA data were reanalyzed with the 1699 gene set used for cross-species clustering. Most of these are cortical admixture to kinase (pink to green).

Supplementary Figure 4C. Heatmap of cross-species clustering analyses for rat adrenal medulla (RAM), RS0 xenograft and RS1/2 xenograft. (C) RS0. Red arrows indicate the positions of the rat samples (blue), which fall within the human-derived pseudohypoxic cluster (orange). Note a small number of human tumors moved from their TCGA-assigned cluster, which was based on 3000 genes, to other clusters when the TCGA data were reanalyzed with the 1699 gene set used for cross-species clustering. Most of these are cortical admixture to kinase (pink to green).

Supplementary Figure 4D. Heatmap of cross-species clustering analyses for rat adrenal medulla (RAM), RS0 xenograft and RS1/2 xenograft. (D) RS1/2. Red arrows indicate the positions of the rat samples (blue), which fall within the human-derived pseudohypoxic cluster (orange). Note a small number of human tumors moved from their TCGA-assigned cluster, which was based on 3000 genes, to other clusters when the TCGA data were reanalyzed with the 1699 gene set used for cross-species clustering. Most of these are cortical admixture to kinase (pink to green).

Supplementary Table 1. Antibodies for immunohistochemistry and immunoblots.

Supplementary Table 2. WGS QC Statistics.

Supplementary Table 3. Effects of post-natal gamma irradiation (5 Gy) on tumor development. Data are expressed as number of tumors/number of animals in which the organs relevant to the type of tumor were examined. The denominators are less than the total numbers of animals in either the non-irradiated or irradiated group because in some cases the relevant organs could not be examined.

Supplementary Table 4. Variant effect predictions.

Supplementary Table 5. Structural variants.

Supplementary Table 6. RNA sequencing data.

## Declaration of interest

The authors declare that there is no conflict of interest that could be perceived as prejudicing the impartiality of the research reported.

## Funding

This research was supported principally by grants to A S T from the SDHB Pheo Para Coalition, the Pheo Para Alliance and the Paradifference Foundation and by the Intramural Research Program of the *Eunice Kennedy Shriver* NICHD, NIH. The work utilized NMR instrumentation purchased from a National Institutes of Health SIG grant (S10OD020073). A D P is supported by the Joseph Herman Trust. R W T is supported by a fellowship from the Victorian Cancer Agency.

## Author contribution statement

J P and A T conceived the study, designed the experiments and wrote the manuscript. B C and X Z performed RNA sequencing and analyzed the data. R T and A D P performed whole genome sequencing and analyzed the data. J D B performed metabolomic experiments and analyzed the data. Y O designed the TALEN. I L and A S-B performed immunohistochemical studies. H D S, S J M, T L and K T S contributed to experiments on hypoxic signaling and redox balance. All authors discussed the results and commented on the manuscript.
