# A xenograft and cell line model of SDH-deficient pheochromocytoma derived from Sdhb+/− rats

**DOI:** 10.1530/ERC-19-0474e

**Published:** 2020-10-01

**Authors:** James F Powers, Brent Cochran, James D Baleja, Hadley D Sikes, Andrew D Pattison, Xue Zhang, Inna Lomakin, Annette Shepard-Barry, Karel Pacak, Sun Jin Moon, Troy F Langford, Kassi Taylor Stein, Richard W Tothill, Yingbin Ouyang, Arthur S Tischler

**Affiliations:** 1 Department of Pathology and Laboratory Medicine, Tufts Medical Center, Tufts University School of Medicine, Boston, Massachusetts, USA; 2 Department of Developmental, Molecular and Chemical Biology, Tufts University School of Medicine, Boston, Massachusetts, USA; 3 Department of Chemical Engineering, Massachusetts Institute of Technology, Cambridge, Massachusetts, USA; 4 Department of Clinical Pathology, University of Melbourne, Melbourne, Victoria, Australia; 5 Section on Medical Neuroendocrinology, Eunice Kennedy Shriver Division National Institute of Child Health and Human Development, Bethesda, Maryland, USA; 6 Peter MacCallum Cancer Centre, Melbourne, Victoria, Australia; 7 Cyagen US Inc, Santa Clara, California, USA

The authors and journal apologise for an error in the above paper, which appeared in volume 27 part 6, pages 337–354. The error relates to the artwork of Table 1 on page 346, in which the units were given in micromoles per milligram of tissue, when the units should have been given in nanomoles per milligram of tissue.

The correct Table 1 is given in full below:

**Table 1:** NMR metabolomic profiles of RS0, RS1/2 xenografts and rat adrenal medulla (RAM).

		RS0 (*n *= 4)		RS1/2 (*n *= 3)	
RAM (20 pooled)			Mean ± s.e.m.		Mean ± s.e.m.
The 10 most abundant detectable metabolites (nmol/mg of tissue)					
Epinephrine	16.15	Lactate	15.18 ± 3.66	Norepinephrine	33.87 ± 29.41
Norepinephrine	12.33	Taurine	12.11 ± 2.69	Lactate	11.45 ± 2.18
Glucose	2.31	**Succinate**	5.99 ± 1.19	Taurine	10.38 ± 0.83
Lactate	0.89	Glycine	3.47 ± 0.75	Ascorbate	7.611 ± .42
Taurine	0.61	Glutamate	2.57 ± 0.58	myo-Inositol	6.33 ± 2.80
ATP (or ADP)	0.59	Ascorbate	2.57 ± 0.64	Glutamate	4.16 ± 0.97
Ascorbate	0.44	Alanine	2.08 ± 0.45	Dopamine	2.55 ± 2.04
AMP	0.30	Creatine	1.39 ± 0.28	O-Phosphoethanolamine	2.06 ± 0.25
ADP (or ATP)	0.21	sn-Glycero-3-phosphocholine	1.29 ± 0.34	AMP	1.85 ± 1.39
Glutamate	0.17	myo-Inositol	1.21 ± 0.26	Betaine	1.83 ± 0.31
**Succinate**				**Succinate**	
30 of 67	0.01			30 of 67	0.29 ± 0.10
Catecholamine and metabolite profile (nmol/mg of tissue)					
Dopamine	0.07		0.38 ± 0.08		2.55 ± 2.04
3,4-Dihydroxybenzeneacetate^a^	0.00		0.05 ± 0.02		0.23 ± 0.05
Norepinephrine	12.33		0.06 ± 0.03		33.87 ± 29.41
Normetanephrine	0.00		0.02 ± 0.01		0.00 ± 0.00
Epinephrine	16.15		0.00 ± 0.00		0.81 ± 0.78
Tyrosine	0.00		0.11 ± 0.01		0.03 ± 0.00

^a^3,4 dihydroxyphenylacetic acid (DOPAC).

