# Supplementary material for: A xenograft and cell line model of SDH-deficient pheochromocytoma derived from Sdhb+/− rats
Source: Endocr Relat Cancer. 2020 Apr 3;27(6):337–54. doi: 10.1530/ERC-19-0474 (PMC7219221; doi:10.1530/ERC-19-0474)
Supplement: Supplementary Figure 1. The TALEN design for Sdhb disruption. The Sdhb gene is located on rat chromosome 5. Exon 1 was selected as the TALEN target site. TALEN mRNA was generated by in vitro transcription, which was then injected into fertilized eggs for KO rat production. The founders were genotype [file supplementary_figure_1.pdf]

Supplementary Figure 1 The TALEN design for *Sdhb* disruption

Sequence and locus of the 13 bp TALEN insertion, pairwise comparison to wild type rat DNA

Pairwise Sequence Comparison

Wildtype	5’-cggggtctccttgaagcgcggcttttcggctacggctctcggcagagtcggcctgcaggtgagccccggagc-3’
(-13)	5’-cggggtctccttgaagcgcggcttttcggc-------------agagtcggcctgcaggtgagccccggagc-3’

Supplementary Figure 1 The *Sdhb* gene is located on rat chromosome 5. Exon 1 was selected as the TALEN target site. TALEN mRNA was generated by *in vitro* transcription, which was then injected into fertilized eggs for KO rat production. The founders were genotyped by PCR followed by DNA sequencing analysis. The mRNA transcribed from the targeted allele with frameshift undergoes nonsense mediated decay.
