# Supplementary figures and images for: A xenograft and cell line model of SDH-deficient pheochromocytoma derived from Sdhb+/− rats
Source: Endocr Relat Cancer. 2020 Apr 3;27(6):337–54. doi: 10.1530/ERC-19-0474 (PMC7219221; doi:10.1530/ERC-19-0474)

**A**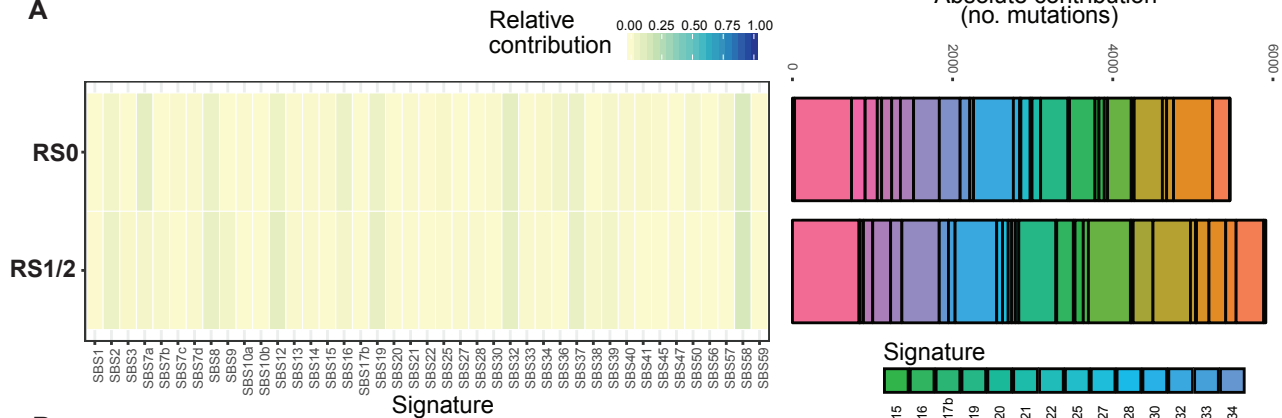**B**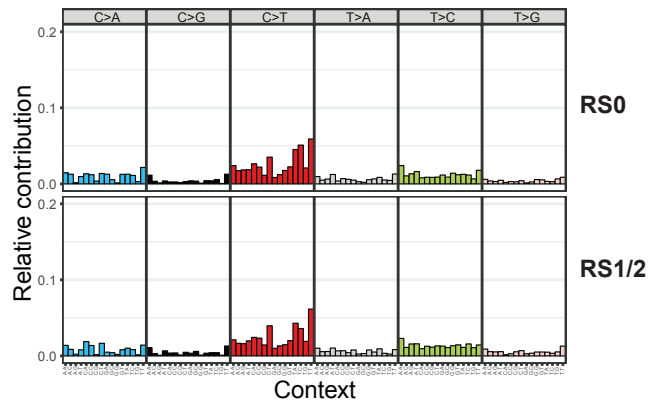

Supplement: Supplementary Figure 2. (A) Mutation signatures of RS0 and RS1/2. The coding region of rat was determined to be 37.48 Mb. RS0 had a mutational load of 30/37.48 = 0.8004269 muts/Mb. RS1/2 had a mutational load of 36/37.48 = 0.96 muts/Mb. Mutation signatures were generated against both models using Mu [file supplementary_figure_2.pdf]

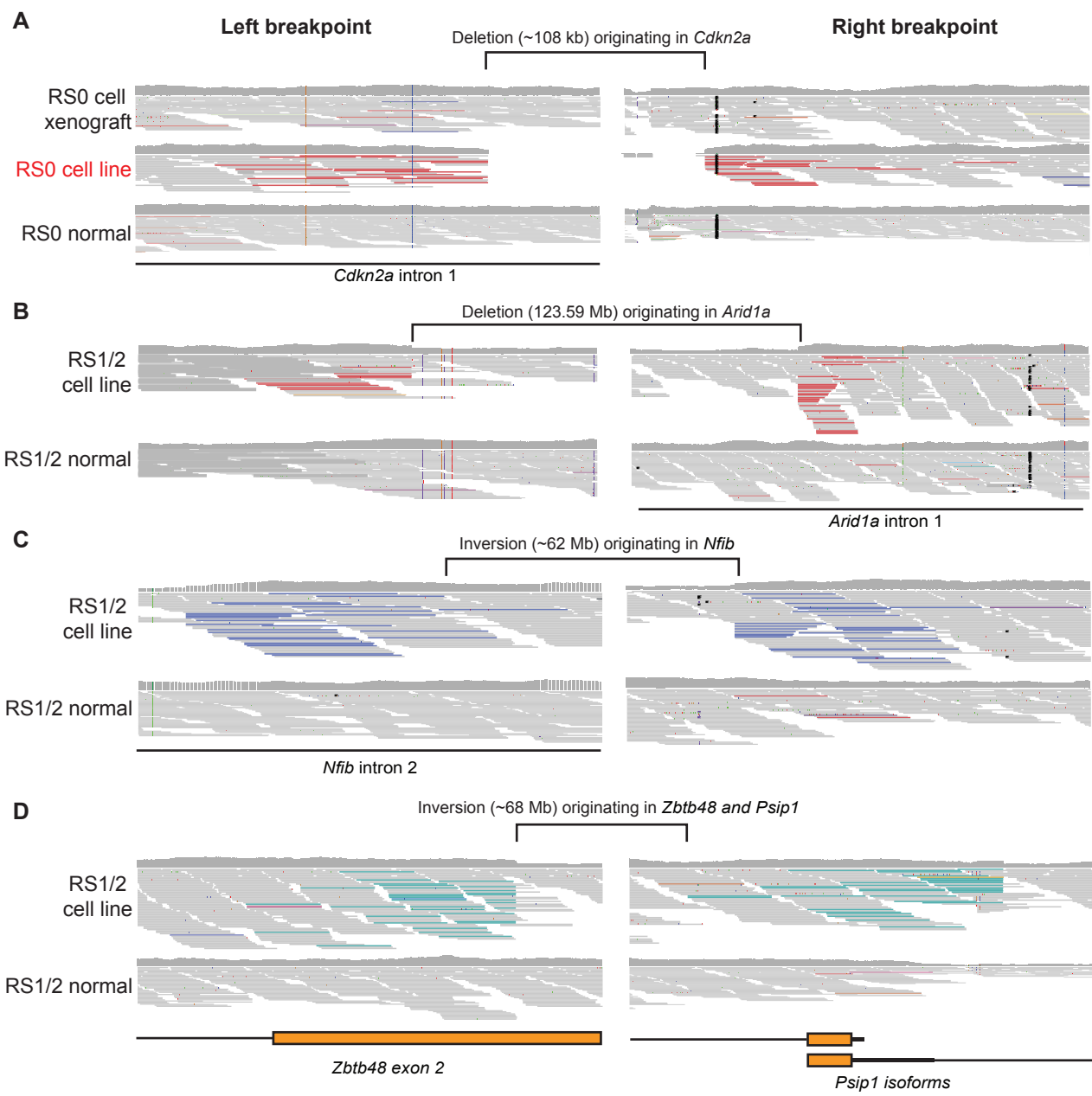

Supplement: Supplementary Figure 3. Genome browser snapshot of sequence coverage involving breakpoints spanning a ~108,205bp deletion encompassing exon 1 of Cdkn2A in cell line RS0. Top: RS0 xenograft; Middle: RS0 cell line; Bottom: matching normal adrenal. Mate-pair reads spanning breakpoints left (chr5:107827 [file supplementary_figure_3.pdf]

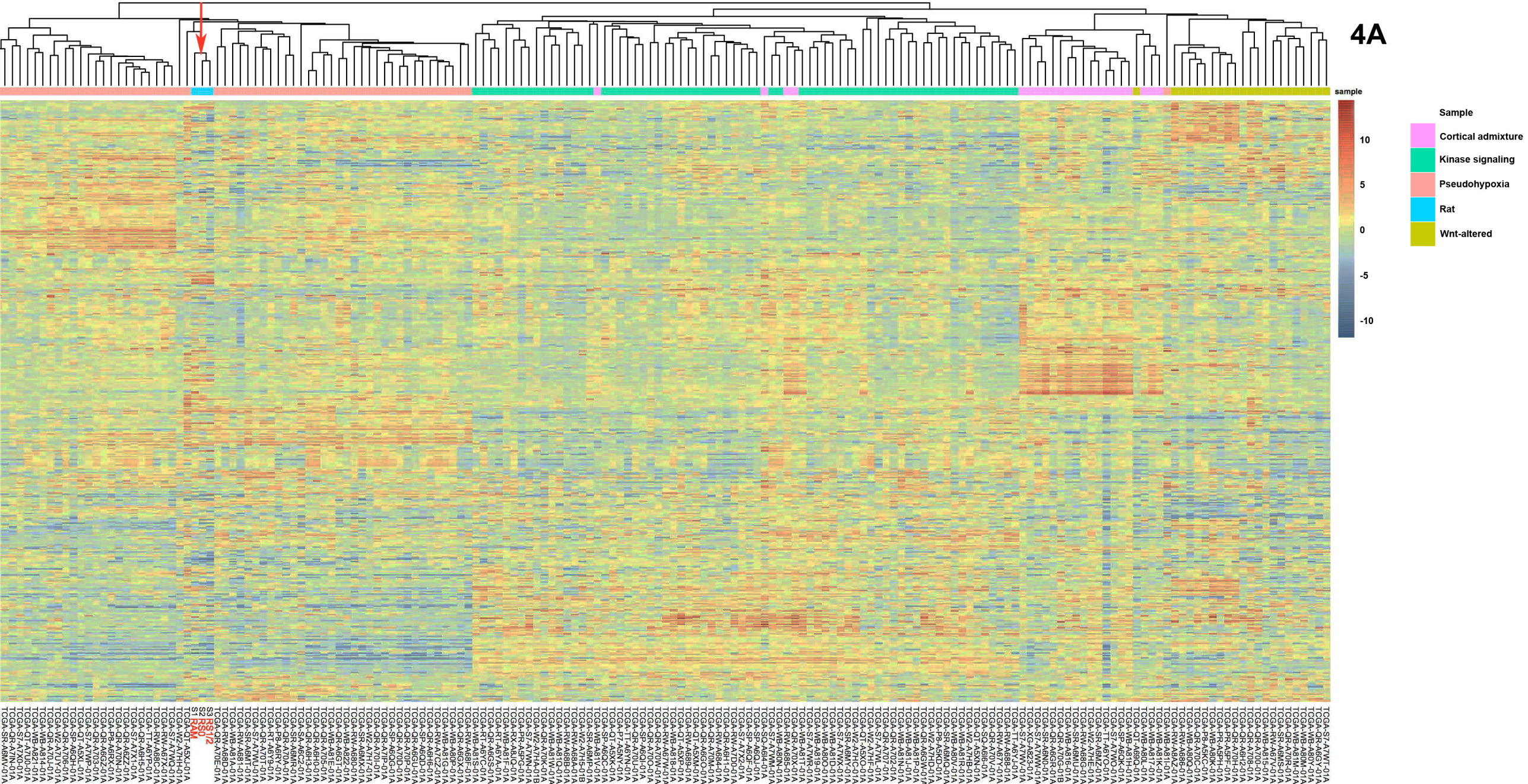

Supplement: Supplementary Figure 4A. Heatmap of cross-species clustering analyses for rat adrenal medulla (RAM), RS0 xenograft and RS1/2 xenograft. (A) All 3 rat samples. Red arrows indicate the positions of the rat samples (blue), which fall within the human-derived pseudohypoxic cluster (orange). Note a small [file supplementary_figure_4A.pdf]

**4B**

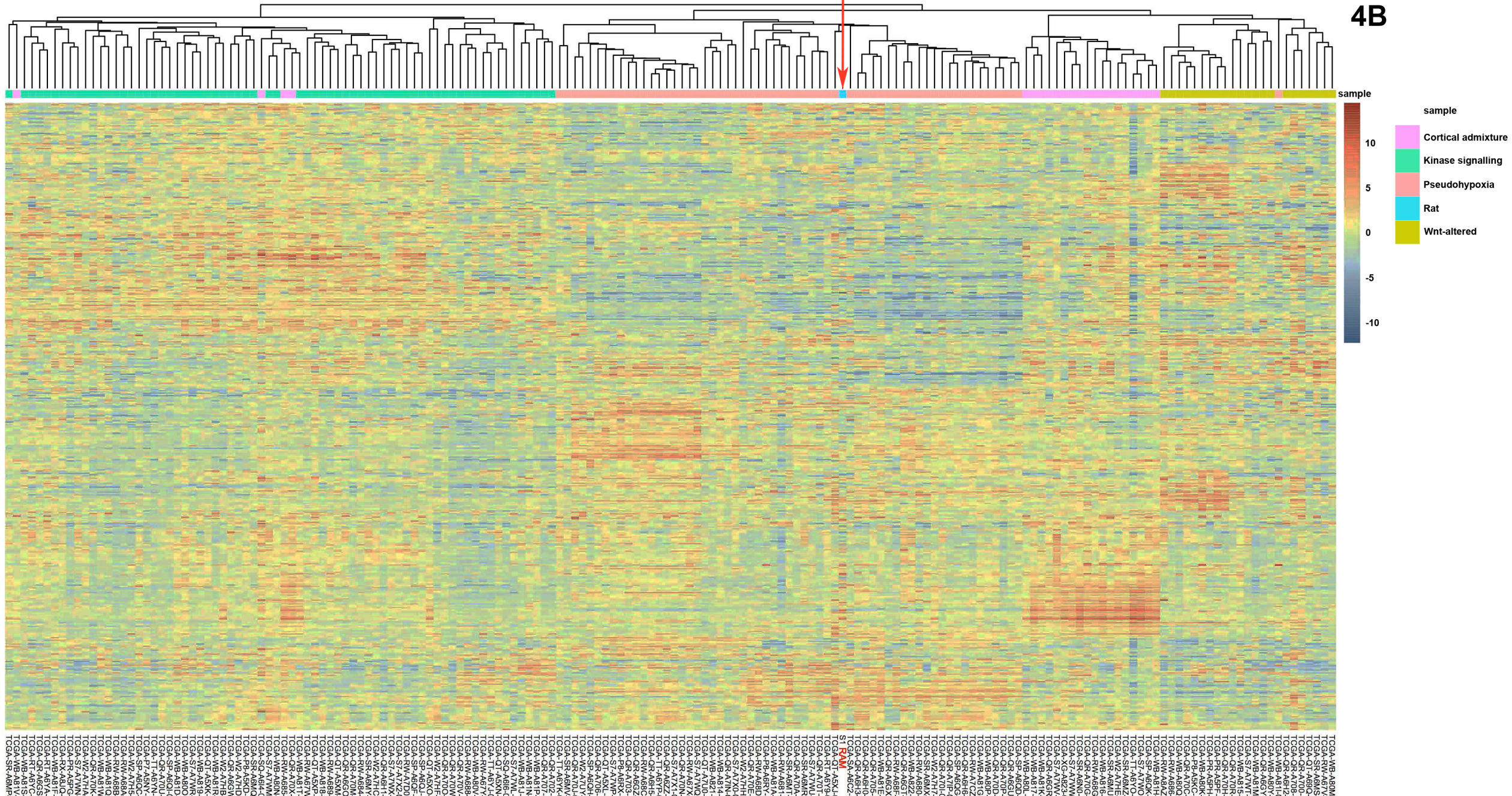

Supplement: Supplementary Figure 4B. Heatmap of cross-species clustering analyses for rat adrenal medulla (RAM), RS0 xenograft and RS1/2 xenograft. (B) RAM. Red arrows indicate the positions of the rat samples (blue), which fall within the human-derived pseudohypoxic cluster (orange). Note a small number of hum [file supplementary_figure_4B.pdf]

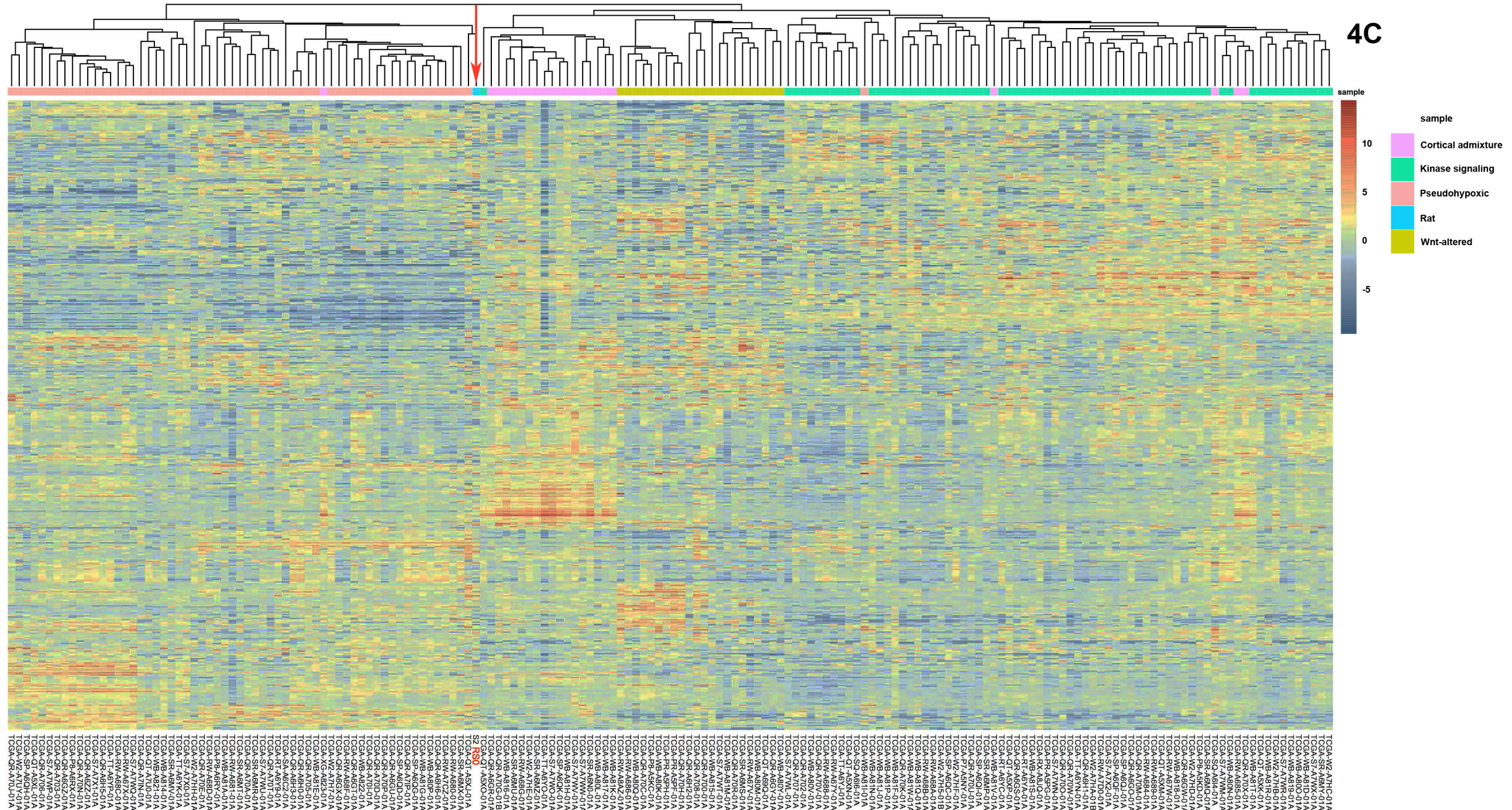

Supplement: Supplementary Figure 4C. Heatmap of cross-species clustering analyses for rat adrenal medulla (RAM), RS0 xenograft and RS1/2 xenograft. (C) RS0. Red arrows indicate the positions of the rat samples (blue), which fall within the human-derived pseudohypoxic cluster (orange). Note a small number of hum [file supplementary_figure_4C.pdf]

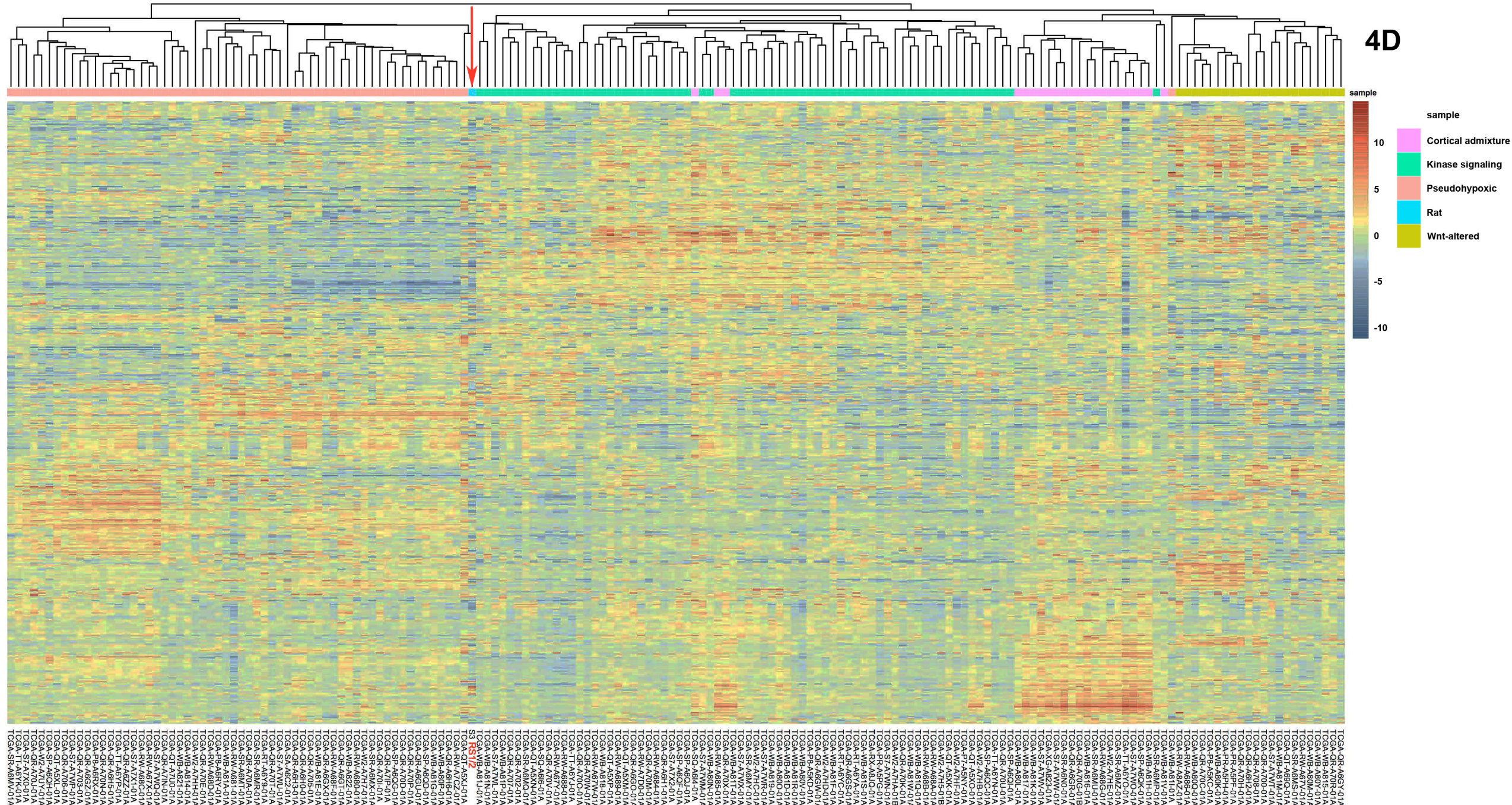

Supplement: Supplementary Figure 4D. Heatmap of cross-species clustering analyses for rat adrenal medulla (RAM), RS0 xenograft and RS1/2 xenograft. (D) RS1/2. Red arrows indicate the positions of the rat samples (blue), which fall within the human-derived pseudohypoxic cluster (orange). Note a small number of h [file supplementary_figure_4D.pdf]
