# Supplementary material for: A xenograft and cell line model of SDH-deficient pheochromocytoma derived from Sdhb+/− rats
Source: Endocr Relat Cancer. 2020 Apr 3;27(6):337–54. doi: 10.1530/ERC-19-0474 (PMC7219221; doi:10.1530/ERC-19-0474)
Supplement: Supplementary Table 1. Antibodies for immunohistochemistry and immunoblots. [file supplementary_table_1.pdf]

Supplementary Data

Table S1 Antibodies for immunohistochemistry and immunoblots

Antibody	Source	Catalogue #	Dilution
BrdU	Becton-Dickinson	347580	1:100
SDHA	Abcam	Ab14715	1:250
SDHB	Sigma	HPA002868	1:2000
Tyrosine hydroxylase	Immunostar	22941	1:4000
Actin (C-11)	Santa Cruz	SC-1615	1:2000
Chromogranin A (GE19)	Gift from R. Fischer-Colbrie.	(Marksteiner et al. Neuroscience 104(2):325-333 2001	1:6000
HIF2 alpha (EPAS1)	Bethyl Laboratories	A700-003	1:1000
