# Supplementary material for: A xenograft and cell line model of SDH-deficient pheochromocytoma derived from Sdhb+/− rats
Source: Endocr Relat Cancer. 2020 Apr 3;27(6):337–54. doi: 10.1530/ERC-19-0474 (PMC7219221; doi:10.1530/ERC-19-0474)
Supplement: Supplementary Table 3. Effects of post-natal gamma irradiation (5 Gy) on tumor development. Data are expressed as number of tumors/number of animals in which the organs relevant to the type of tumor were examined. The denominators are less than the total numbers of animals in either the non-irradiat [file supplementary_table_3.pdf]

Table S3 Effects of post-natal gamma irradiation (5 Gy) on tumor development. Data are expressed as number of tumors/number of animals in which the organs relevant to the type of tumor were examined. The denominators are less than the total numbers of animals in either the non-irradiated or irradiated group because in some cases the relevant organs could not be examined.

	Non-irradiated<br># of tumors/# of animals (%)	Irradiated<br># of tumors/# of animals (%)
Pheochromocytoma		
Macro	1/13 (7.7)	3/16 (18.7)
Micro	4/13 (30.7)	6/16 (37.5)
Carotid PG	0/10	1/9 (11.1)
Pituitary Adenoma	3/13 (23.1)	7/12 (58.3)
